# Variance‐Guided Regression for Heteroscedastic Data With a Grouping‐Based Extension for Nonlinear Prediction

**DOI:** 10.1002/sim.70632

**Published:** 2026-06-07

**Authors:** Sibei Liu, Min Lu

**Affiliations:** ^1^ Division of Biostatistics Miller School of Medicine, University of Miami Miami Florida USA

**Keywords:** heteroscedasticity, high dimensionality, iteratively reweighted algorithms, lagrangian optimization, nonlinearity

## Abstract

Although homoscedasticity is often assumed in linear regression, real data may show variance patterns or residual structures that violate this assumption. We propose VarGuid, a variance‐guided framework for two related settings: Covariate‐dependent conditional variance under a global linear mean model, and residual nonlinear mean structure that can mimic heteroscedasticity. The framework has two deliberately separated components. The first uses an iteratively reweighted regression (IRR) algorithm to estimate a sparse global linear mean–variance model and support coefficient interpretation. The second uses a biconvex artificial‐grouping algorithm for conditional prediction, keeping the fitted linear backbone fixed while adding group‐specific local intercept corrections. We establish predictive‐risk guarantees for the global estimator, and simulations and empirical studies show improved out‐of‐sample accuracy. VarGuid is illustrated in two applications: Health‐related quality of life in low‐ and middle‐income countries, and high‐dimensional genomic prediction of lymph node evaluation in breast cancer.

## 1 Introduction

Although homoscedasticity is often assumed in linear models, this assumption is frequently violated in the analysis of real‐world data [1, 2]. When this occurs, existing methods typically take one of two paths. One path retains the mean model and focuses on valid uncertainty quantification under heteroscedasticity through robust inference procedures [3, 4, 5, 6]. The other path models the variance or dispersion explicitly through heteroscedastic regression, variance‐function or log‐variance regression, and double generalized linear models [7, 8, 9, 10]. These approaches address different statistical goals. In practice, however, a single dataset may raise two distinct questions: Whether the outcome has covariate‐dependent variance within an otherwise linear mean model, and whether residual patterns that appear heteroscedastic are instead signals of nonlinear mean misspecification.

**FIGURE 1:**
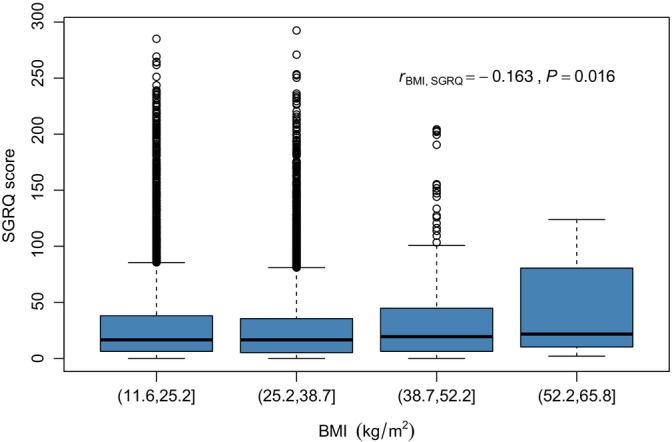
Relationship between BMI and SGRQ score in LMICs. The association estimation may be affected by heteroscedasticity due to violating the homoscedasticity assumption (Breusch Pagan test statistic = 62.32, P<0.001).

For example, in the study by Siddharthan et al. [11] on lung condition in low‐income and middle‐income countries (LMICs), Body Mass Index (BMI) appears related not only to the median *St. George's Respiratory Questionnaire (SGRQ)* score but also to its variability, as illustrated in Figure 1. The SGRQ is a validated patient‐reported instrument that measures health‐related quality of life in individuals with chronic respiratory disease. While individuals with higher BMI values show a higher median SGRQ score, the overall correlation between BMI and SGRQ is significantly negative, contrary to the generally reported positive association between BMI and SGRQ in related settings [12, 13, 14]. It is therefore unclear whether the negative association in these LMIC data reflects context‐specific biology, model misspecification, or a failure of the homoscedasticity assumption.

For data patterns such as Figure 1, one plausible explanation is that the response follows a global linear mean structure while its variability also depends on the predictors. This motivates the heteroscedastic regression model studied in Section 2. Suppose we observe independent data $\left({\text{X}}_{i},Y_{i}\right)$, i=1,2,…,n, where ${\text{X}}_{i}={\left(1,X_{i2},\ldots ,X_{ip}\right)}^{⊤}$ contains the predictor variables and $Y_{i}$ represents the response. The first column of the design matrix is always a column of 1s corresponding to the intercept. We do not assume homogeneity as in the usual regression setup. In general, we write

(1) $$Y_{i}={\text{X}}_{i}^{⊤}\beta +\left({\text{X}}_{i}^{⊤}\gamma \right)\varepsilon_{i},$$

where $\varepsilon_{i}$ are i.i.d. with $𝔼(\varepsilon_{i})=0$ and $\mathrm{Var}(\varepsilon_{i})=\sigma^{2}$, $\beta ={\left(\beta_{1},\ldots ,\beta_{p}\right)}^{T}$ denotes the coefficients for the conditional mean of the response, and $\gamma ={\left(\gamma_{1},\ldots ,\gamma_{p}\right)}^{T}$ governs the variance index. Note that if ${\text{X}}_{i}^{⊤}\gamma =1$ with $\gamma_{2}=\cdots =\gamma_{p}=0$, the above model reduces to the usual homoscedastic regression setup. Equation (1) defines the global linear mean–variance quasi‐likelihood model studied in Section 2.

At the same time, apparent heteroscedasticity need not reflect a true variance mechanism. Because standard heteroscedasticity diagnostics are applied to residuals from a fitted linear mean model, a nonlinear mean structure can generate residual patterns that appear heteroscedastic even when the underlying error variance is constant. This motivates a second, distinct task: Improving point prediction when the global linear mean model in Equation (1) is not fully adequate.

The paper, therefore, contains two connected but conceptually distinct components. Section 2 is estimation‐oriented: It fits a sparse global linear heteroscedastic model, jointly estimates $\left(\hat{\beta },\hat{\gamma }\right)$, and supports interpretation of the global regression coefficients in $\hat{\beta }$. When no penalization is applied to the mean coefficients, approximate standard errors for $\hat{\beta }$ can be obtained from the final weighted least‐squares step. Section 3, by contrast, is a conditional prediction extension built on the fitted linear backbone from Section 2. It keeps $\hat{\beta }$ from the first stage fixed and adds grouping‐based local intercept adjustments to absorb residual nonlinear mean structure. This second stage is not a Bayesian formulation and does not propagate first‐stage uncertainty; its role is to improve out‐of‐sample point prediction rather than to provide a second layer of coefficient inference.

Related heteroscedastic and joint mean–variance/dispersion approaches already establish separate modeling of the mean and variance/dispersion, and iterative reweighting [7, 8, 9, 10]. Against this background, our contributions are threefold. First, in Section 2, we study a penalized joint mean–variance quasi‐likelihood estimator for heteroscedastic regression in which both β and γ may be parse. The contribution here is the specific penalized formulation, its adaptation to high‐dimensional settings, and the accompanying predictive‐risk theory for the resulting coupled loss. Second, in Section 3, we introduce a distinct grouping‐based extension that uses residual structure from the fitted linear model to absorb remaining nonlinear mean effects while retaining the global linear backbone given by $\hat{\beta }$. Third, in Section 4, we evaluate these two components in low‐ and high‐dimensional medical examples, separating coefficient interpretation under the global mean–variance model from out‐of‐sample prediction using the grouping‐based extension. Section 5 concludes, and additional material is provided in the appendices.

To support reproducibility and implementation, we provide an open‐source R package, varGuid, that implements the proposed estimator and the artificial‐grouping prediction extension as a two‐phase analysis: The first phase for coefficient estimation and the second phase for conditional prediction.

## 2 The Variance Guided (VarGuid) Coefficient Estimator

### 2.1 General Loss Function

For Equation (1), we define the following per‐observation quasi‐loss:

(2) $$\begin{array}{cc} \ell (y,\text{x};\beta ,\gamma ) & =\frac{{\left(y-{\text{x}}^{⊤}\beta \right)}^{2}}{2\;{\left({\text{x}}^{⊤}\gamma \right)}^{2}}+\log|{\text{x}}^{⊤}\gamma |,\;\text{well‐defined if}\;|{\text{x}}^{⊤}\gamma |>0. \end{array}$$

Write the *population risk* R(β,γ)=𝔼ℓ(Y,X;β,γ) and the *empirical risk* $R_{n}(\beta ,\gamma )=\frac{1}{n}\sum_{i=1}^{n}\ell (Y_{i},{\text{X}}_{i};\beta ,\gamma )$. Our estimator minimizes the penalized empirical risk

$$\hat{\beta },\hat{\gamma }\in \arg\underset{\beta ,\gamma }{\min}\;{𝒬}_{n}(\beta ,\gamma ):=R_{n}(\beta ,\gamma )+\lambda_{\beta }\|\beta {\|}_{1}+\lambda_{\gamma }\|\gamma {\|}_{1}.$$

This estimator reduces to weighted least squares when the penalty terms vanish (i.e., $\lambda_{\beta }=\lambda_{\gamma }=0$), where the first term adaptively weights squared residuals by the inverse local variance ${\left({\text{X}}_{i}^{⊤}\gamma \right)}^{-2}$ and the second term, $\log|{\text{X}}_{i}^{⊤}\gamma |$, enforces identifiability of the variance scale and arises naturally in variance modeling. This loss function coincides with the negative log‐likelihood under Gaussian errors, but more importantly, it remains valid as a quasi‐likelihood or M‐estimation criterion whenever the errors have finite variance [15]. In this sense, it is a very general objective function that yields consistent quasi‐likelihood estimates whenever the conditional variance is linear in form and the errors have finite variance.

### 2.2 Theoretical Guarantees

In this section, we establish that, at the population level, the heteroscedastic model class underlying our estimator achieves predictive quasi‐risk that is no worse (and typically strictly better) than the homoscedastic Lasso baseline. We also provide a finite‐sample oracle inequality under high‐dimensional sparsity. Let $\text{X}={\left[{\text{X}}_{1}^{⊤},\ldots ,{\text{X}}_{n}^{⊤}\right]}^{⊤}$ denote the n×p design matrix built from the observation‐level covariate vectors ${\text{X}}_{i}$.

Assumption 1 (Regularity) Suppose that ${\left(Y_{i},{\text{X}}_{i}\right)}_{i=1}^{n}$ are i.i.d. with $Y_{i}\in \mathbb{R}$ and ${\text{X}}_{i}\in {\mathbb{R}}^{p}$, and that:

i. $𝔼\|{\text{X}}_{i}{\|}_{2}^{2}<\infty$ and $𝔼(Y_{i}^{2})<\infty$.
ii. The conditional error $\varepsilon_{i}$ satisfies $𝔼(\varepsilon_{i})=0$, $\mathrm{Var}(\varepsilon_{i})=1$ and $𝔼(\varepsilon_{i}^{4})<\infty$.
iii. For all (β,γ) under consideration, $|{\text{X}}_{i}^{⊤}\gamma |\geq c_{\eta }>0$ almost surely (to avoid division by zero).
iv. The design matrix X satisfies a restricted eigenvalue condition of order $s=s_{\beta }+s_{\gamma }$, where $s_{\beta }=\|\beta^{⋆}{\|}_{0}$ and $s_{\gamma }=\|\gamma^{⋆}{\|}_{0}$ are the sparsities of the oracle pair defined below.

Assumption 1 is standard in high‐dimensional M‐estimation: (i)–(ii) impose finite moments on the data and innovations, (iii) rules out degenerate variance indices, and (iv) provides a restricted eigenvalue condition required for oracle rates. The next result formalizes that, at the population level, allowing covariates to drive heteroscedasticity can only improve predictive quasi‐risk relative to a homoscedastic baseline.

Proposition 1 (Population quasi‐risk dominance) *Let*

$$\begin{array}{cc} \left(\beta^{⋆},\gamma^{⋆}\right) & \in \arg\underset{\beta ,\gamma }{\min}R(\beta ,\gamma ),\;(\beta^{\mathrm{hom}},\gamma^{\mathrm{hom}})\in \arg\underset{\beta ,\gamma :\;x^{⊤}\gamma \equiv c>0}{\min}R(\beta ,\gamma ). \end{array}$$

*Under Assumption* *1*, *we have*

$$R(\beta^{⋆},\gamma^{⋆})\leq R(\beta^{\mathrm{hom}},\gamma^{\mathrm{hom}}),$$

*with strict inequality whenever the covariate‐specific optimal scale* $\eta^{⋆}(x)\in \arg\min_{u>0}𝔼\;\left[\frac{{\left(Y-x^{⊤}\beta^{⋆}\right)}^{2}}{2u^{2}}+\log u|{\text{X}}_{0}=x\right]$ *is nonconstant on a set of positive probability, where* $\left(Y,{\text{X}}_{0}\right)$ *denotes a generic observation*.

Proposition 1 motivates modeling the variance index explicitly. We now provide finite‐sample guarantees for the penalized estimator. The following oracle inequality shows that our penalized estimator achieves the usual (slogp)/n prediction error rate under sparsity.

Theorem 1 (Oracle inequality in high dimensions) *Let* $\left(\hat{\beta },\hat{\gamma })\in \arg\min{𝒬}_{n}(\beta ,\gamma \right)$, *where*

$${𝒬}_{n}(\beta ,\gamma )=\frac{1}{n}\overset{n}{\underset{i=1}{\sum }}\ell (Y_{i},{\text{X}}_{i};\beta ,\gamma )+\lambda_{\beta }\|\beta {\|}_{1}+\lambda_{\gamma }\|\gamma {\|}_{1}.$$

*Suppose Assumption* *1* *holds*, $\lambda_{\beta },\lambda_{\gamma }≍\sqrt{\frac{\log p}{n}}$, *and* $\left(\beta^{⋆},\gamma^{⋆})=\arg\min_{\beta ,\gamma :\;\|\beta {\|}_{0}\leq s_{\beta },\;\|\gamma {\|}_{0}\leq s_{\gamma }}R(\beta ,\gamma \right)$ *is the oracle sparse pair. Then with probability at least* 1−Cexp(−clogp),

$$R(\hat{\beta },\hat{\gamma })-R(\beta^{⋆},\gamma^{⋆})\;≲\;(s_{\beta }+s_{\gamma })\;\frac{\log p}{n},$$

*where* c,C>0 *are universal constants*.

As shown in Supporting Information, the proof of Proposition 1 follows directly from nesting arguments, while the proof of Theorem 1 combines concentration inequalities, restricted eigenvalue conditions, and decomposability of the $\ell_{1}$ penalty [16, 17]. In particular, a standard symmetrization plus concentration argument shows that

$$\underset{\beta ,\gamma }{\sup}|\frac{1}{n}\overset{n}{\underset{i=1}{\sum }}\ell (Y_{i},{\text{X}}_{i};\beta ,\gamma )-R(\beta ,\gamma )|=O_{p}\left(\sqrt{\frac{\log p}{n}}\right),$$

which provides the uniform stochastic bound needed in the oracle inequality.

Remark 1 Proposition 1 and Theorem 1 together show that the proposed estimator achieves the same predictive risk as homoscedastic Lasso when the variance is constant, and strictly better predictive quasi‐risk when the variance depends on covariates. Moreover, in high‐dimensional regimes, the excess risk relative to the oracle sparse pair decays at the usual (slogp)/n rate. This provides theoretical justification for using 𝒬(β,γ) in place of the standard Lasso.

#### 2.2.1 Novelty of Our Results

Classical heteroscedastic and joint mean–variance/dispersion approaches, including heteroscedastic linear models, variance‐function/log‐variance regression, double generalized linear models, and iterative/adaptive weighted estimation, already establish the broader idea of separate modeling of the mean and variance/dispersion [7, 8, 10, 18]. The general high‐dimensional machinery for penalized M‐estimators with decomposable regularizers has been developed in prior work [16, 17]. We therefore do not claim novelty for either ingredient in isolation. Our contribution is twofold. First, Proposition 1 is new: It establishes, at the population level, that the heteroscedastic quasi‐likelihood model strictly extends the homoscedastic Lasso baseline, and that allowing variance covariates can only improve predictive quasi‐risk. Second, Theorem 1 adapts the general oracle inequality framework to the specific penalized joint mean–variance quasi‐likelihood in Equation (2), which is not covered by existing results. In particular, our loss couples mean and variance indices and contains nonstandard terms ${\left({\text{X}}_{i}^{⊤}\gamma \right)}^{-2}$ and $\log|{\text{X}}_{i}^{⊤}\gamma |$. Verifying restricted strong convexity and concentration for this nonconvex risk is technically nontrivial, and our analysis shows that the usual (slogp)/n prediction rate still holds.

### 2.3 Iteratively Reweighted Regression (IRR) Algorithm

Direct minimization of 𝒬(β,γ) is difficult due to the nonconvexity in γ and the nonsmooth $\ell_{1}$ penalties. To address this, we employ a block‐coordinate descent strategy that alternates between updating γ and updating β. The γ‐update is carried out using a Newton–Raphson step combined with coordinate‐wise soft‐thresholding to enforce sparsity, while the β‐update is performed by solving a weighted Lasso problem given the current estimate of γ. This alternating procedure is iterated until convergence, and the full algorithm is summarized in Algorithm 1. For the variance coefficients γ, define the score vector $u={\left(u_{1},\ldots ,u_{n}\right)}^{⊤}$, where

$$u_{i}=\frac{1}{\eta_{i}}-\frac{r_{i}^{2}}{\eta_{i}^{3}},\;\eta_{i}={\text{X}}_{i}^{⊤}\gamma ,\;r_{i}=Y_{i}-{\text{X}}_{i}^{⊤}\beta .$$

The gradient is then $g={\text{X}}^{⊤}u.$ Similarly, define the curvature vector $d={\left(d_{1},\ldots ,d_{n}\right)}^{⊤}$, where $d_{i}=1/\eta_{i}^{2}-3r_{i}^{2}/\eta_{i}^{4}.$ The resulting Hessian takes the form $\text{H}={\text{X}}^{⊤}\mathrm{diag}(d)\text{X},$ where diag(d) is the n×n diagonal matrix with ith diagonal entry $d_{i}$. To incorporate the $\ell_{1}$ penalty on γ, each coordinate is updated by soft‐thresholding, leading to the Lasso Newton–Raphson step. To ensure numerical stability, we enforce $|\eta_{i}|\geq \epsilon$ with a small constant ϵ>0. For the mean coefficients β, given the current variance estimate, we solve a weighted least‐squares problem with weights $w_{i}=1/\eta_{i}^{2}$; the $\ell_{1}$ penalty on β yields a weighted Lasso problem, solvable with standard routines. By iterating these two block updates until convergence, we obtain the joint estimates $\left(\hat{\beta },\hat{\gamma }\right)$. In implementation, $T_{\text{out}}$ and $T_{\gamma }$ are maximum iteration caps used only as safeguards; the algorithm stops earlier once the successive β and γ updates fall below prespecified tolerances. The penalties $\lambda_{\beta }$ and $\lambda_{\gamma }$ are selected by K‐fold cross‐validation on the training data.

When $\lambda_{\beta }=0$, β is updated by ordinary least squares, and standard errors for $\hat{\beta }$ can be obtained from the corresponding weighted least squares estimator using the weights from the final iteration. Simulation studies on the performance of coefficient estimation are reported in Appendix B of the Supporting Information. Table S2 highlights a critical distinction in the performance of confidence interval estimation methods when heteroscedasticity is addressed through VarGuid compared to methods relying on heteroskedasticity‐robust standard errors for OLS point estimates. Specifically, the confidence interval coverage results for the Sandwich estimator often show notably higher values than the nominal 95% level, indicating a phenomenon of over‐coverage. While sandwich standard errors are valuable for robust inference when the conditional variance structure is not explicitly modeled or is misspecified, our simulations suggest that when a method like VarGuid achieves improved point estimation by explicitly addressing heteroscedasticity, the use of sandwich estimators may lead to over‐coverage of the confidence intervals. This highlights the importance of aligning the variance estimation strategy with the quality and efficiency of the point estimators, potentially favoring methods like weighted least squares‐based standard errors, derived from the explicitly modeled variance structure within VarGuid, for more accurate confidence interval coverage.^1^

ALGORITHM 1 **Joint estimation of γ (Lasso Newton–Raphson) and β (iteratively reweighted ${\text{Lasso}}^{\left[1\right]}$).**

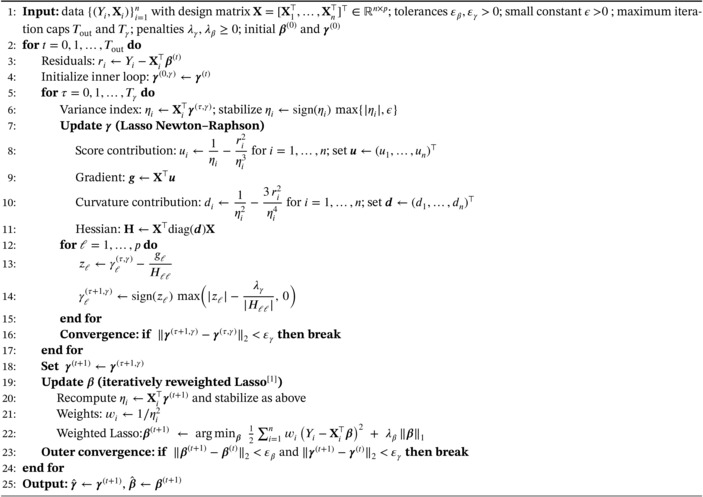

## 3 Conditional Nonlinear Prediction With Artificial Grouping Effects

Section 2 estimates a global linear heteroscedastic model and yields $\left(\hat{\beta },\hat{\gamma }\right)$. In practice, however, residual patterns flagged as heteroscedasticity after the first stage may arise from nonlinear misspecification of the conditional mean rather than from a true variance mechanism. Residual‐based heteroscedasticity diagnostics implicitly assume a correctly specified linear mean function; when this assumption fails, nonlinear relationships can induce residual patterns that mimic heteroscedasticity even when the underlying error variance is constant. This section, therefore, has a different goal from Section 2: It is a conditional prediction extension built on the fitted linear backbone $\hat{\beta }$, not a second inferential model for (β,γ). Throughout this section, $\hat{\beta }$ from Section 2 is treated as fixed. The artificial grouping step estimates only the grouping structure and the associated local intercept adjustments; it does not re‐estimate the global coefficient vector or account for the estimation uncertainty in $\hat{\beta }$. In particular, this stage is not a Bayesian formulation.

If diagnostics suggest a specific parametric revision of the mean model, such as adding a known quadratic term or other prespecified basis expansion, then it is reasonable to enlarge the design matrix and re‐run Algorithm 1. Section 3 is intended for a different setting: The global linear backbone remains useful, but the residual nonlinear component is not specified a priori. In that setting, we keep $\hat{\beta }$ fixed for three reasons. First, doing so preserves the global linear estimand from Section 2 and the approximate Section 2 inference for $\hat{\beta }$ when $\lambda_{\beta }=0$. Second, it avoids confounding between the global linear term ${\text{X}}_{i}^{⊤}\hat{\beta }$ and the adaptive group‐specific intercept correction introduced later in this section; if both were updated jointly, the artificial groups could absorb signal that would otherwise be attributed to the global coefficients, thereby changing their interpretation. Third, conditional on the fixed offset ${\text{X}}_{i}^{⊤}\hat{\beta }$, the grouping step is a simpler conditional optimization, whereas alternating re‐estimation of (β,γ) together with the artificial groups would define a different and more nonconvex model. We do not study that joint scheme here, and we do not currently have corresponding convergence or inferential guarantees for it. Moreover, once a local mean correction is introduced, re‐estimating γ simultaneously would blur whether the remaining residual structure is being attributed to nonlinear mean misspecification or to heteroscedasticity.

**FIGURE 2:**
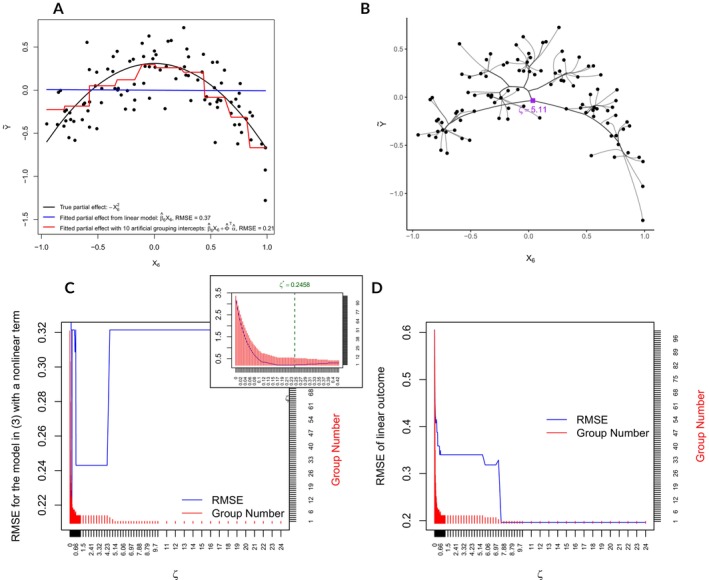
(A) Partial‐effect illustration from Equation (3). The vertical axis represents the partial outcome ${\tilde{Y}}_{i}=Y_{i}-\sum_{j=1}^{5}X_{ij}$ plotted against $X_{i6}$; the red step function shows the artificial‐grouping local intercept correction added to the fitted linear partial effect. (B) Path of subgroup centers as ζ increases; larger ζ yields more subgroup fusion. The horizontal axis shows the subgroup center corresponding to $X_{i6}$, and the vertical axis shows the group mean of $\tilde{Y}$ within each artificial group, included for visual context only. As ζ increases from 0 to 5.11, the centers merge progressively, reducing from n distinct groups to a single group. (C) Test‐set RMSE from Panel **A** as a function of ζ, used to determine the tuning parameter and the corresponding number of artificial groups. The vertical marker indicates the cross‐validated choice of $\zeta^{*}=0.2458$, yielding ten groups and the improved prediction shown by the red line in Panel **A**; the upper‐right inset provides a magnified view of the RMSE curve in this region. When multiple values of ζ yield essentially identical RMSE, ties are resolved in favor of the larger ζ, which produces fewer artificial groups and a more parsimonious solution. (D) Same analysis as in Panel **C** but under a linear outcome, for which the RMSE curve favors a large value of ζ and returns a single‐group solution. Supporting Information: Figure S1 provides the corresponding expanded linear‐case illustration, including the fitted curve, subgroup‐center fusion path, and RMSE trajectory.

To visualize the grouping mechanism while preserving a meaningful linear backbone, consider the partially linear nonlinear model

(3) $$Y_{i}=\overset{5}{\underset{j=1}{\sum }}X_{ij}-X_{i6}^{2}+\varepsilon_{i},$$

where $X_{ij}\overset{\text{iid}}{∼}\text{Unif}(-1,1)$ and $\varepsilon_{i}\overset{\text{iid}}{∼}𝒩(0,0.2)$. If a linear model is fitted in $\left(X_{i1},\ldots ,X_{i6}\right)$, the first five effects are correctly represented by the linear backbone, whereas the nonlinear contribution of $X_{i6}$ is omitted. In Figure 2A, we therefore plot the partial outcome

$${\tilde{Y}}_{i}=Y_{i}-\overset{5}{\underset{j=1}{\sum }}X_{ij}$$

against $X_{i6}$, so that the displayed target is the residual nonlinear partial effect $-X_{i6}^{2}$. In practice, the analogous quantity is the residual $Y_{i}-{\text{X}}_{i}^{⊤}\hat{\beta }$ from the full fitted linear model. This example is used only to visualize the grouping mechanism. More generally, the target setting of Section 3 is one in which a global linear backbone remains useful for interpretation, but residual nonlinear mean structure remains to be corrected for prediction. Residual‐vs.‐fitted plots and standard heteroscedasticity diagnostics applied after the misspecified linear fit can flag such structure; in this example, the apparent residual pattern is caused by omitted nonlinear mean structure rather than by nonconstant error variance.

In this section, we use residual structure from the first‐stage linear fit to construct artificial local intercept adjustments. Unlike the IRR algorithm in Section 2, which jointly estimates the global coefficients in (β,γ), the present stage does not update $\hat{\beta }$ or $\hat{\gamma }$. Instead, the fitted linear predictor ${\text{X}}_{i}^{⊤}\hat{\beta }$ is retained as a fixed offset and is augmented by a local correction shared by observations assigned to the same artificial group. The use of artificial random effects was illustrated by Rao et al. [19], where synthetic effects were used to obtain local predictions for new data points that fall outside the range of the training data. Here, the grouping variable is not tied to a hierarchical sampling structure but is generated synthetically from covariate and residual structure to create local adjustments for both training and test points. The resulting predictor combines a global effect from the observed variables through $\hat{\beta }$, which is common to all observations, with an artificial local effect shared only within the same data‐adaptive group. In the partial‐effect illustration of Figure 2A, this local correction can substantially reduce prediction error relative to an ordinary linear fit.

For multivariate covariates, we need to determine the grouping variable systematically. Denote the subgroup‐center matrix by

$$U={\left[u_{1}^{⊤},\ldots ,u_{n}^{⊤}\right]}^{⊤}\in {\mathbb{R}}^{n\times p},$$

where $u_{i}\in {\mathbb{R}}^{p}$ is the center attached to observation i. Individuals i and j belong to the same artificial group if $u_{i}=u_{j}$. The quantity $u_{i}$ is not a scientific parameter of direct interest; it is a data‐adaptive center in predictor space used only to induce artificial groups. For p=1, $u_{i}$ is a scalar location on the predictor axis. For p=2, $u_{i}$ is a point in the two‐dimensional predictor plane. More generally, for p predictors, $u_{i}\in {\mathbb{R}}^{p}$, and the fusion penalty operates on Euclidean distances $\|u_{i}-u_{j}{\|}_{2}$ in this space. Let $c_{1},\ldots ,c_{q}$ denote the q distinct rows of U. The prediction model is a fixed‐effect model based on the observed covariates X and the artificial groups, which introduce group‐specific intercepts,

(4) $$\hat{\text{Y}}=\text{X}\hat{\beta }+\Phi \alpha ,$$

where $\Phi =(\phi_{ij})$ is an n×q artificial grouping design matrix, with $\phi_{ij}=1\{u_{i}=c_{j}\}$. Here, Equation (4) is written in vector form: $\hat{Y}\in {\mathbb{R}}^{n}$, $\text{X}\in {\mathbb{R}}^{n\times p}$, $\hat{\beta }\in {\mathbb{R}}^{p}$, $\Phi \in {\mathbb{R}}^{n\times q}$, and $\alpha \in {\mathbb{R}}^{q}$. Equivalently, at the observation level, ${\hat{Y}}_{i}={\text{X}}_{i}^{⊤}\hat{\beta }+\phi_{i}^{⊤}\alpha$. After ζ is selected by cross‐validation in Algorithm 2, the final prediction uses the selected quantities $\hat{\Phi }$ and $\hat{\alpha }$. After fusion, each observation belongs to exactly one artificial group, so each row of Φ contains a single entry equal to 1 and all remaining entries equal to 0. The vector $\alpha ={\left(\alpha_{1},\ldots ,\alpha_{q}\right)}^{⊤}$ contains group‐specific intercepts and satisfies $\sum_{j=1}^{q}\alpha_{j}=0$. Equivalently, write $m({\text{X}}_{i})=𝔼(Y_{i}|{\text{X}}_{i})$ for the conditional mean at observation i. Then the grouped term $\phi_{i}^{⊤}\alpha$ can be viewed as a piecewise‐constant approximation to the residual mean $m({\text{X}}_{i})-{\text{X}}_{i}^{⊤}\hat{\beta }$, with the partition learned through supervised fusion. The parameter $\hat{\beta }$ is obtained from Algorithm 1, and α and Φ are given through the following supervised extension of Chi and Lange's clustering method [20]:

(5) $$\begin{array}{cc} & \underset{\alpha ,U}{\text{minimize}}\;\frac{1}{2}\overset{n}{\underset{i=1}{\sum }}{\left(Y_{i}-{\text{X}}_{i}^{⊤}\hat{\beta }-\phi_{i}^{⊤}\alpha \right)}^{2}+\frac{1}{2}\overset{n}{\underset{i=1}{\sum }}{\left\|{\text{X}}_{i}-u_{i}\right\|}_{2}^{2}+\zeta \underset{i<j}{\sum }w_{ij}{\left\|u_{i}-u_{j}\right\|}_{2},\\ & \text{subject to}\;\overset{q}{\underset{j=1}{\sum }}\alpha_{j}=0. \end{array}$$

where ζ is a positive tuning constant and $w_{ij}=w_{ji}=f({\text{X}}_{i},{\text{X}}_{j})$ is a nonnegative weight derived from Euclidean distance or an external model such as a random forest proximity matrix [21, 22]. When ζ is large, all the subgroups are merged together, that is, $u_{1}=u_{2}=\cdots =u_{n}$, so q=1, $\Phi ={\text{1}}_{n}$, and the constraint forces α=0. Equation (5) then reduces to the standard linear predictor without artificial grouping effects. When ζ=0, the minimum value is achieved when each $u_{i}={\text{X}}_{i}$, implying that each point belongs to its own group. As ζ increases, the centers of these groups begin to merge; see Figure 2B for illustration.

### 3.1 The Lagrangian Function

To derive the Lagrangian form of Equation (5), we handle two parts separately. One part addresses the constraint $\sum_{j=1}^{q}\alpha_{j}=0$, and the other handles the fusion penalty $\zeta \sum_{i<j}w_{ij}{\left\|u_{i}-u_{j}\right\|}_{2}.$ Importantly, $\hat{\beta }$ is not optimized in this stage; it enters only through the fixed linear predictor from Section 2. The unknown quantities are the local intercepts α and the center matrix U. An auxiliary matrix V is introduced below to impose the pairwise difference constraints.

Let $\lambda_{0}>0$ be a quadratic penalty weight for the constraint $\sum_{j=1}^{q}\alpha_{j}=0$. Define

$${\tilde{\text{Y}}}_{\hat{\beta }}=\left[\begin{array}{l} \text{Y}-\text{X}\hat{\beta }\\ 0 \end{array}\right],\;\tilde{\Phi }(\lambda_{0})=\left[\begin{array}{l} \Phi \\ \sqrt{\lambda_{0}}{\text{1}}_{q}^{⊤} \end{array}\right].$$

Since the constraint $\sum_{j=1}^{q}\alpha_{j}=0$ is equivalent to ${\left\{\sum_{j=1}^{q}\alpha_{j}\right\}}^{2}/2=0$, the local‐intercept part can be written as

(6) $$\frac{1}{2}\overset{n}{\underset{i=1}{\sum }}{\left(Y_{i}-{\text{X}}_{i}^{⊤}\hat{\beta }-\phi_{i}^{⊤}\alpha \right)}^{2}+\frac{\lambda_{0}}{2}{\left(\overset{q}{\underset{j=1}{\sum }}\alpha_{j}\right)}^{2}=\frac{1}{2}{\left\|{\tilde{\text{Y}}}_{\hat{\beta }}-\tilde{\Phi }(\lambda_{0})\alpha \right\|}_{2}^{2}.$$

For the fusion penalty $\zeta \sum_{i<j}w_{ij}{\left\|u_{i}-u_{j}\right\|}_{2}$, we introduce a new L×p auxiliary matrix V, where L=n2, so that the optimization can alternate between U and V. For pair l=(i,j) with i<j, denote $w_{l}=w_{ij}$, and let $d_{l}$ be an n‐dimensional row vector with $d_{li}=1$, $d_{lj}=-1$, and all other entries equal to zero. Then $d_{l}U=u_{i}-u_{j}.$ We define $v_{l}$, the lth row of V, as an auxiliary copy of this pairwise difference and impose the constraint

$$v_{l}=u_{i}-u_{j}=d_{l}U.$$

Therefore, $\text{minimize}\;\;\frac{1}{2}\sum_{i=1}^{n}{\left\|{\text{X}}_{i}-u_{i}\right\|}_{2}^{2}+\zeta \sum_{i<j}w_{ij}{\left\|u_{i}-u_{j}\right\|}_{2}$ can be equivalently written as

$$\begin{array}{cc} \text{minimize}\; & \frac{1}{2}\overset{n}{\underset{i=1}{\sum }}{\left\|{\text{X}}_{i}-u_{i}\right\|}_{2}^{2}+\zeta \overset{L}{\underset{l=1}{\sum }}w_{l}{\left\|v_{l}\right\|}_{2}\\ \text{subject to}\; & d_{l}U-v_{l}=\text{0},\;l=1,\ldots ,L. \end{array}$$

The constraints are enforced through quadratic penalties $\frac{1}{2}{\left\|d_{l}U-v_{l}\right\|}_{2}^{2}$. The corresponding contribution is

(7) $$\frac{1}{2}\overset{n}{\underset{i=1}{\sum }}{\left\|{\text{X}}_{i}-u_{i}\right\|}_{2}^{2}+\zeta \overset{L}{\underset{l=1}{\sum }}w_{l}{\left\|v_{l}\right\|}_{2}+\overset{L}{\underset{l=1}{\sum }}\frac{\lambda_{l}}{2}{\left\|d_{l}U-v_{l}\right\|}_{2}^{2}.$$

Combining Equations (6) and (7) gives

(8) $$\begin{array}{cc} ℒ(\alpha ,U,V,\lambda_{0},\lambda_{1}) & =\frac{1}{2}{\left\|{\tilde{\text{Y}}}_{\hat{\beta }}-\tilde{\Phi }(\lambda_{0})\alpha \right\|}_{2}^{2}+\frac{1}{2}\overset{n}{\underset{i=1}{\sum }}{\left\|{\text{X}}_{i}-u_{i}\right\|}_{2}^{2}\\ & \;+\zeta \overset{L}{\underset{l=1}{\sum }}w_{l}{\left\|v_{l}\right\|}_{2}+\overset{L}{\underset{l=1}{\sum }}\frac{\lambda_{l}}{2}{\left\|d_{l}U-v_{l}\right\|}_{2}^{2}, \end{array}$$

where $\lambda_{1}={\left(\lambda_{1},\lambda_{2},\ldots ,\lambda_{L}\right)}^{⊤}$ contains positive quadratic penalty weights for enforcing the pairwise fusion constraints.

### 3.2 The Biconvex Algorithm

The challenge in minimizing Equation (8) is that Φ depends discretely on U, so the problem is not jointly differentiable in all unknown quantities. Because $\hat{\beta }$ is fixed from Algorithm 1, the optimization alternates between the local‐intercept block α and the grouping block $G={\left[U^{⊤},V^{⊤}\right]}^{⊤}$. When updating α, G and Φ are held fixed. When updating G, α and Φ are held fixed. Finally, Φ is updated after G is updated. In iteration m+1, the local‐intercept update is

(9) $$\alpha^{\left(m+1\right)}(\lambda_{0}):=\underset{\alpha }{\text{argmin}}\;\frac{1}{2}{\left\|{\tilde{Y}}_{\hat{\beta }}-\tilde{\Phi }(\lambda_{0})\alpha \right\|}_{2}^{2}.$$

We then choose

(10) $$\lambda_{0}^{\left(m+1\right)}=\underset{\lambda_{0}}{\text{argmin}}\;\frac{1}{2}{\left\|{\tilde{\text{Y}}}_{\hat{\beta }}-\tilde{\Phi }(\lambda_{0})\alpha (\lambda_{0})\right\|}_{2}^{2},\;\alpha^{\left(m+1\right)}=\alpha \left(\lambda_{0}^{\left(m+1\right)}\right).$$

Thus, the local‐intercept block updates only α; it does not re‐estimate the global coefficient vector $\hat{\beta }$. This separation preserves the global linear target from Section 2 and prevents the adaptive grouping step from redefining the meaning of $\hat{\beta }$.

For the G part, minimizing Equation (8) with respect to U, while holding the other blocks fixed, yields

(11) $$\begin{array}{cc} u_{i}^{\left(m+1\right)} & =\frac{\begin{array}{l} X_{i}+\sum_{l:d_{li}=1,d_{lk}=-1}\lambda_{l}^{\left(m\right)}(u_{k}^{\left(m\right)}+v_{l}^{\left(m\right)})+\sum_{l:d_{li}=-1,d_{lk}=1}\lambda_{l}^{\left(m\right)}(u_{k}^{\left(m\right)}-v_{l}^{\left(m\right)}) \end{array}}{1+\sum_{l:d_{li}=1}\lambda_{l}^{\left(m\right)}+\sum_{l:d_{li}=-1}\lambda_{l}^{\left(m\right)}}, \end{array}$$

where k denotes the unique partner index paired with i in pair l; that is, $d_{li}=1$ implies $d_{lk}=-1$ for a unique k, and $d_{li}=-1$ implies $d_{lk}=1$ for a unique k. The penalty‐weight update is implemented as the projected step

(12) $$\lambda_{l}^{\left(m+1\right)}=\max\left\{\lambda_{\min},\;\lambda_{l}^{\left(m\right)}-\delta \frac{1}{2}{\left\|d_{l}U^{\left(m\right)}-v_{l}^{\left(m\right)}\right\|}_{2}^{2}\right\},$$

where δ is the step length and $\lambda_{\min}>0$ keeps the denominator in the V‐update bounded away from zero.

For the V block, we solve

(13) $$v_{l}^{\left(m+1\right)}=\underset{v\in {\mathbb{R}}^{p}}{\text{argmin}}\;\zeta \;w_{l}{\left\|v\right\|}_{2}+\frac{\lambda_{l}}{2}{\left\|d_{l}U^{\left(m\right)}-v\right\|}_{2}^{2}.$$

Let $a_{l}^{\left(m\right)}=d_{l}U^{\left(m\right)}$. For pair l=(i,j), $a_{l}^{\left(m\right)}=u_{i}^{\left(m\right)}-u_{j}^{\left(m\right)}\in {\mathbb{R}}^{p}$. Thus, the shrinkage is applied to the full pairwise difference vector rather than separately to each coordinate. The solution is the vector soft‐thresholding update

(14) $$v_{l}^{\left(m+1\right)}=\left\{\begin{array}{ll} \left(1-\frac{\zeta w_{l}}{\lambda_{l}{\left\|a_{l}^{\left(m\right)}\right\|}_{2}}\right)a_{l}^{\left(m\right)},\; & {\left\|a_{l}^{\left(m\right)}\right\|}_{2}>\frac{\zeta w_{l}}{\lambda_{l}},\\ \text{0},\; & {\left\|a_{l}^{\left(m\right)}\right\|}_{2}\leq \frac{\zeta w_{l}}{\lambda_{l}}. \end{array}\right.$$

Algorithm 2 summarizes our procedure, and Line 17 specifies that the tuning parameter ζ is selected by minimizing the cross‐validated RMSE of the outcome, with ties resolved in favor of the largest ζ and hence the fewest artificial groups. In Algorithm 2, M is the maximum number of inner biconvex iterations allowed for each candidate value of ζ. It is used as a computational stopping cap. The algorithm may stop before reaching M when the updates in U and the fitted local correction stabilize. The tuning parameter selected by cross‐validation is ζ, not M. For a new test observation ${\text{X}}_{\text{new}}$, the artificial group is assigned using the fitted subgroup centers from the training data. Specifically, we set

$$\hat{g}({\text{X}}_{\text{new}})=\arg\underset{1\leq j\leq \hat{q}}{\min}\|{\text{X}}_{\text{new}}-{\hat{c}}_{j}{\|}_{2},$$

and use the corresponding local intercept adjustment ${\hat{\alpha }}_{\hat{g}({\text{X}}_{\text{new}})}$. The resulting prediction is

$${\hat{Y}}_{\text{new}}={\text{X}}_{\text{new}}^{⊤}\hat{\beta }+{\hat{\alpha }}_{\hat{g}({\text{X}}_{\text{new}})}.$$

Figure 2C,D illustrate how this selection mechanism determines the number of artificial groups. In the nonlinear setting of Equation (3), the RMSE curve in Figure 2C reaches its minimum at an intermediate value of ζ, corresponding to ten groups and improved prediction relative to a purely linear model. By contrast, when the underlying mean structure is linear, as in Figures 2D and Supporting Information: Figure S1, the RMSE decreases monotonically as ζ increases, and the optimal value yields a single‐group solution. This behavior shows that the method does not create unnecessary local groups and naturally collapses to an ordinary linear regression model when no nonlinear adjustment is needed. Finally, we note that $\hat{\beta }$ from Algorithm 1 remains the global linear component in Equation (4); the artificial grouping mechanism modifies only the local intercepts through $\hat{\alpha }$ and does not alter the global regression coefficients.

ALGORITHM 2 **Generation of artificial grouping effects $\hat{\alpha }$ and $\hat{\Phi }$.**

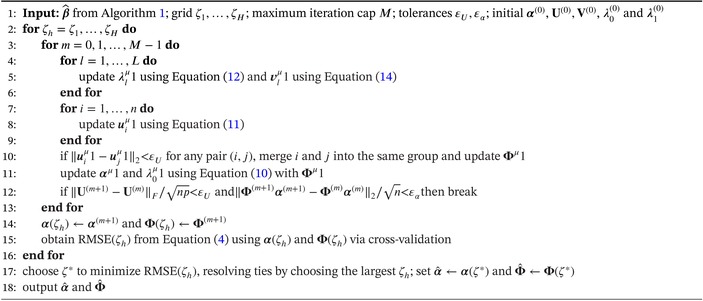

#### 3.2.1 Connection to Mixture of Regressions

Our artificial grouping approach is superficially related to a mixture of regression models [23, 24], in which latent subpopulations are assumed to generate the data, and group memberships are inferred jointly with regression coefficients. The key distinction is that we do not posit a latent mixture distribution or interpret the artificial groups as scientific subpopulations. Instead, we construct artificial groups from the residual structure of the fitted global linear model and use them only as local intercept corrections for conditional prediction. Thus, α and Φ are predictive adjustment parameters rather than population‐level coefficients analogous to β or γ. This yields a supervised, optimization‐based adjustment that improves prediction while retaining a single global regression backbone given by $\hat{\beta }$, rather than estimating separate regression coefficients for latent components.

### 3.3 Empirical and Simulation Results for Model Prediction

#### 3.3.1 Purpose of the Prediction Comparisons

The comparisons in this subsection are limited to out‐of‐sample point prediction. The machine‐learning methods are included as predictive benchmarks, not as competitors for coefficient‐level inference or uncertainty quantification. The interpretable component of VarGuid is the global linear backbone $\hat{\beta }$ estimated in Section 2. The grouped extension in Section 3 keeps this backbone fixed and adds only the local correction ${\hat{\phi }}_{i}^{⊤}\hat{\alpha }$ for prediction. Consequently, RMSE‐based comparisons in this subsection should be interpreted as evaluating the conditional prediction extension, not as evidence of improved uncertainty quantification.

This subsection evaluates whether the prediction extension of VarGuid from Algorithm 2 enhances model prediction, using both empirical and simulated datasets for the analysis. As shown in Table 1, we have 11 real datasets with n>p from the University of California, Irvine Machine Learning Repository (UCI), and 10 high‐dimensional datasets with p>n from the datamicroarray Github R package [25]. All the high‐dimensional datasets have categorical outcomes, such as cancer type. To construct a continuous outcome for each dataset, we first applied the Lasso model [26] to predict the categorical outcome using all standardized gene expressions. We then selected the gene expression with the highest absolute coefficient value as the outcome Y. The remaining gene expressions were used as predictors X. We also developed 10 simulated nonlinear scenarios to test prediction performance, with details provided in Appendix C of the Supporting Information.

**TABLE 1:** Real‐world datasets with low‐ and high‐dimensional features for evaluating outcome prediction performance.

Datasets	Source^a^	n ^b^	p ^b^	Outcome	References
Concrete	UCI	1030	9	Concrete compressive strength	Yeh [27]
Liver Disorders	UCI	345	5	Drinks	Forsyth [28]
Airfoil Self‐Noise	UCI	1503	5	Scaled sound pressure	Brooks and Marcolini [29]
Real Estate Valuation	UCI	414	4	House price in New Taipei City, Taiwan	Yeh [30]
Average Localization Error	UCI	107	4	ALE^c^ in sensor node localization process	Singh and Lee [31]
Auto MPG	UCI	398	7	MPG	Quinlan [32]
Concrete Slump Test	UCI	103	7	Compressive strength	Yeh [33]
Yacht Hydrodynamics	UCI	308	6	Residuary resistance	Gerritsma and Versluis [34]
Servo	UCI	167	4	Class from a simulation of a servo system	Ulrich [35]
Demand forecasting orders	UCI	60	12	Total orders	Ferreira et al. [36]
Facebook metrics	UCI	500	18	Total interactions	Moro and Vala [37]
Alon	DM	62	2000	X765	Alon et al. [38]
Christensen	DM	217	1413	OSMP188F	Christensen et al. [39]
Gravier	DM	168	2905	g1CNS26	Gravier, Eleonore et al. [40]
Pomeroy	DM	60	7128	D28473‐s‐at	Pomeroy et al. [41]
Shipp	DM	58	6817	V2006	Shipp et al. [42]
Singh	DM	102	12 600	V10234	Singh et al. [43]
Tian	DM	173	12 625	898sat	Tian et al. [44]
West	DM	49	7 129	V132	Wei et al. [45]
Gordon	DM	181	12 533	34320at	Gordon and Olshen [46]
Subramanian	DM	50	10 100	BAX	Subramanian et al. [47]

^a^ UCI stands for the University of California, Irvine Machine Learning Repository; DM stands for the datamicroarray Github R package. For DM datasets, the outcome column contains the names of genes that serve as the dependent variable Y.

^b^ n stands for number of samples, p for number of variables.

^c^ ALE stands for Average Localization Error.

For the low‐dimensional cases, we configured the simulations with N=500 and p=15. For the high‐dimensional cases, we set N=100 with p=200. These simulations included both independent and correlated feature scenarios with correlation ρ=0.9. For all benchmark datasets, we randomly selected 80% of the data for training the model and 20% for evaluating prediction accuracy, conducting 100 replications in each case.

For the low‐dimensional scenarios, we compared VarGuid with two mixture‐model competitors: The “regmixEM” procedure, a finite mixture regression model implemented in the mixtools R package [48], and the “flexMix” model‐based clustering framework from the flexmix package [49]. For the high‐dimensional scenarios, we benchmarked VarGuid against Lasso‐based estimation, where the tuning parameters $\lambda_{\beta }$ and $\lambda_{\gamma }$ in Algorithm 1 were selected by a grid search using 10‐fold cross‐validation, and the Lasso penalty parameter λ for the baseline model was chosen using 10‐fold cross‐validation in the glmnet package [50]. We additionally evaluated the FMRS method [51] implemented in the fmrs package [52]. For both low‐ and high‐dimensional settings, we further compared performance against several machine‐learning regression approaches, including Random Forests, Gradient Boosting, XGBoost, LightGBM, CatBoost, and Bayesian Additive Regression Trees (BART), all tuned using standard cross‐validation defaults for their respective R packages.

**FIGURE 3:**
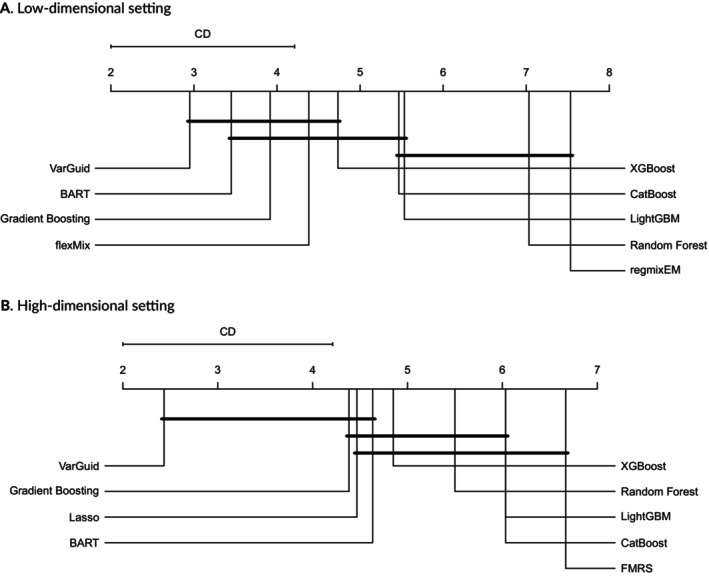
Critical difference diagram of average method ranks for RMSE comparison for outcome prediction on real and simulated datasets.

For the low‐dimensional simulation settings, Figure 3A displays the critical difference (CD) diagram summarizing the average ranks of all methods based on their RMSE across the simulation scenarios. Each method's mean rank is shown along the horizontal axis, with lower values indicating superior performance. A horizontal bar connecting two or more methods denotes a group that is not significantly different according to the Nemenyi test at the specified significance level. In these settings, VarGuid attains the lowest average rank, followed by Gradient Boosting, XGBoost, and Random Forest, whereas the mixture‐model approaches flexMix and regmixEM exhibit substantially poorer performance. The CD line at the top of the figure indicates the minimum difference in average rank required for two methods to be declared significantly different; differences smaller than this threshold are not statistically distinguishable.

Figure 3B shows the corresponding CD diagram for the high‐dimensional simulation settings. VarGuid again achieves the best overall rank, clearly outperforming both penalized regression (Lasso) and mixture‐model estimation (FMRS). Among the machine‐learning baselines, Gradient Boosting and XGBoost forms the next tier of competitive performers, followed by Random Forest, LightGBM, CatBoost, and BART. Methods joined by a horizontal bar constitute a group without significant differences under the Nemenyi test. As in Figure 3A, the CD line at the top marks the minimum separation in average rank required for a statistically significant difference; smaller separations indicate that the methods cannot be differentiated statistically. Details on the method implementation and additional information related to Figure 3 are provided in Appendix C of the Supporting Information.

These results, therefore, support the use of artificial grouping as a post‐estimation prediction correction when the residual nonlinear structure remains after fitting the global linear model. They should not be interpreted as showing that the grouped extension provides coefficient‐level uncertainty quantification. Formal inference for the grouped correction, including propagation of the first‐stage uncertainty in $\hat{\beta }$ and $\hat{\gamma }$, is outside the scope of the present work.

## 4 Data Applications

We evaluate VarGuid using two real‐world health datasets that differ substantially in dimensionality. Unless otherwise stated, the data applications in this section use the estimator from Algorithm 1. The artificial grouping procedure in Algorithm 2 is evaluated separately in Section 3 as a prediction‐oriented extension and is not used for the coefficient estimates reported below. Section 4.1 examines respiratory‐related quality of life in LMICs, and Section 4.2 analyzes high‐dimensional gene expression profiles from the PAM50 study [53] for predicting lymph node involvement in breast cancer patients.

### 4.1 Exploring Factors Related to SGRQ Scores in LMICs

We first analyze determinants of SGRQ scores, a validated measure of respiratory health burden, in the LMIC dataset of Siddharthan et al. [11] introduced in Figure 1. The dataset includes 10 664 participants from semi‐urban Bhaktapur (Nepal), urban Lima (Peru), and rural Nakaseke (Uganda), and captures demographic, clinical, and environmental predictors such as age, sex, education, biomass fuel use, body mass index (BMI), smoking, heart disease, tuberculosis, and diabetes.

All predictors were standardized to allow direct comparison of effect sizes. Table 2 shows the estimated associations with SGRQ scores. The strongest predictors of worse respiratory quality of life were heart disease (β=27.70, SE =2.69, p<0.001), tuberculosis (β=14.08, SE =1.87, p<0.001), and current smoking (β=3.99, SE =1.10, p<0.001). Tuberculosis remains a major contributor to long‐term respiratory impairment in LMICs, consistent with prior evidence on its lasting impact on lung function and quality of life [54].

**TABLE 2:** Estimated regression coefficients for SGRQ score and related factors.

	Estimate	Std. Error	t statistic	p
Intercept	37.27	22.82	1.63	0.102
Biomass use	−2.15	0.94	−2.29	0.022
Sex (female)	−4.28	0.83	−5.18	<0.001
Education	−1.08	0.10	−10.85	<0.001
Age	1.01	22.67	0.04	0.964
BMI^a^	−0.11	0.08	−1.35	0.178
Current smoker	3.99	1.10	3.63	<0.001
Heart disease	27.70	2.69	10.31	<0.001
Tuberculosis	14.08	1.87	7.53	<0.001
Diabetes	4.89	1.56	3.13	0.002

^a^ For BMI, the VarGuid bivariate correlation was −0.012 (SE=0.012, p=0.321), compared with the OLS estimate r=−0.163 (SE=0.068, p=0.016) in Figure 1.

From an effect‐estimation perspective, the dominant associations with worse SGRQ are heart disease, tuberculosis, and current smoking, all of which are clinically plausible predictors of respiratory health burden. The BMI result is especially informative. The crude BMI–SGRQ display in Figure 1 shows a negative marginal association together with visible changes in spread, whereas the adjusted VarGuid mean coefficient for BMI is small and not statistically significant (β=−0.11, p=0.178). This attenuation suggests that, in this cohort, BMI is more strongly related to variability in respiratory burden than to a monotone shift in mean SGRQ score. Thus, the LMIC application illustrates the estimation‐oriented component of VarGuid: Coefficient interpretation is based on the global linear mean model, while the variance‐guided weighting helps avoid overinterpreting a crude association that is entangled with heteroscedasticity.

### 4.2 Predicting Lymph Node Evaluation From Gene Expression Data

We next apply VarGuid with iteratively reweighted Lasso to RNA‐seq data from the PAM50 study [53], focusing on predicting the number of axillary lymph nodes examined, a clinically important indicator of disease severity and surgical assessment. This application is intended as a high‐dimensional prediction and variable‐selection example. The selected genes are therefore interpreted as predictors selected by the penalized fitting procedure, not as causal drivers.

We first evaluated predictive performance using only the 50 PAM50 genes. Under 10‐fold cross‐validation, Lasso achieved an RMSE of 8.74, whereas VarGuid reduced the RMSE to 7.65, indicating improved prediction within the PAM50 set. All RMSE comparisons for VarGuid in this subsection use both Algorithm 1 and the artificial grouping extension from Algorithm 2.

**FIGURE 4:**
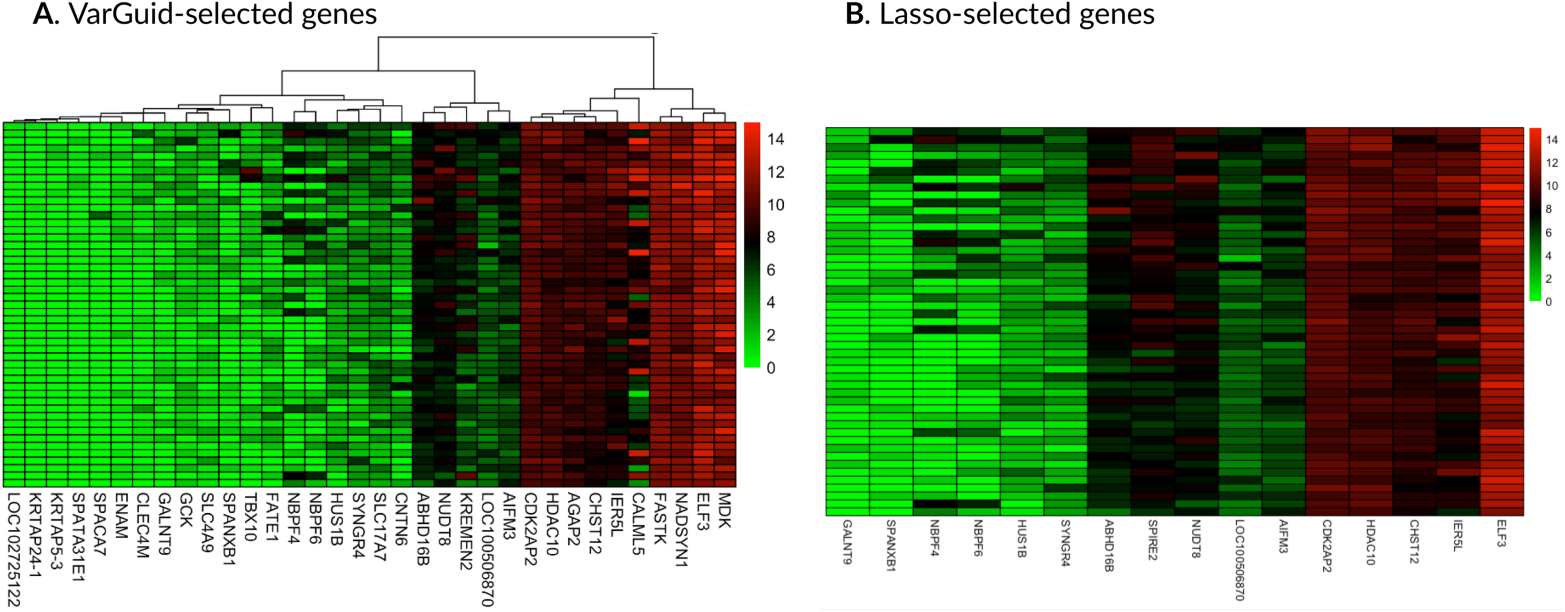
Gene expression heatmaps for breast cancer samples (n=50), ordered by the number of axillary lymph nodes examined. Panel A: 34 genes selected by VarGuid from p=20133. Panel B: 16 genes selected by Lasso. All Lasso‐selected genes were included in the VarGuid model except *SPIRE2*. No PAM50 genes overlapped with the selected sets.

We then expanded the analysis to all p=20133 available genes. With all genes included, Lasso achieved an RMSE of 7.118, while VarGuid further reduced it to 7.108. VarGuid selected 34 genes (Figure 4A) compared to 16 genes selected by Lasso (Figure 4B); the Lasso‐selected genes were a subset of those chosen by VarGuid, except for *SPIRE2*. No PAM50 genes were selected by either method, consistent with previous findings that PAM50 subtypes, while prognostically useful, do not strongly predict the extent of lymph node evaluation [55, 56].

These results illustrate that while PAM50 is clinically informative for molecular subtyping, it is not sufficiently predictive for lymph node evaluation. Expanding the feature space improves predictive accuracy, and VarGuid provides a more comprehensive selection of relevant genes while maintaining competitive RMSE. The modest RMSE difference between VarGuid and Lasso in the all‐gene analysis indicates that the two methods perform comparably when the full gene set is available. This application is intended primarily as a variable selection and prediction example in a high‐dimensional genomic setting, rather than as an investigation of conditional variance mechanisms.

## 5 Conclusions

This paper studies two related but distinct consequences of heteroscedasticity signals in linear regression. First, when the conditional variance itself depends on covariates, Section 2 considers a sparse global linear heteroscedastic model and estimates the mean coefficients β and variance‐index coefficients γ jointly through a penalized quasi‐likelihood. Second, when residual patterns flagged as heteroscedasticity are instead driven by the nonlinear mean misspecification, Section 3 introduces a separate grouping‐based correction built on the fitted linear predictor from Section 2. This two‐component framing clarifies that VarGuid is an estimation‐oriented procedure for a global linear mean–variance model, followed by a conditional prediction extension for residual nonlinearity.

The roles of the two stages are different. Section 2 is estimation‐oriented: It provides an interpretable global linear backbone through $\hat{\beta }$ and $\hat{\gamma }$, and when $\lambda_{\beta }=0$, approximate model‐based standard errors for $\hat{\beta }$ can be obtained from the final weighted least‐squares step. Section 3 is prediction‐oriented: It keeps $\hat{\beta }$ fixed and adds artificial group‐specific intercept adjustments to improve out‐of‐sample point prediction when linearity in the conditional mean is inadequate. This second stage is not a Bayesian formulation and does not propagate first‐stage uncertainty; rather, it is a supervised conditional prediction device designed to preserve the interpretability of the global linear component while allowing local nonlinear corrections.

The artificial grouping term has a specific statistical interpretation in this framework. It is not intended to represent latent scientific subpopulations or a random‐effects distribution. Instead, it provides a piecewise‐constant approximation to residual mean structure left unexplained by the global linear fit. Equivalently, the grouped term estimates local intercept corrections around the backbone predictor ${\text{X}}_{i}^{⊤}\hat{\beta }$. This interpretation explains why the grouped extension can improve prediction without replacing the single global linear model or estimating separate regression coefficients for different latent classes.

Across simulations and applications, the target of evaluation follows this separation. In the LMIC application, Section 2 is used to estimate and interpret associations under a global heteroscedastic linear model. In the nonlinear simulations and prediction benchmark studies, Section 3 is used to evaluate out‐of‐sample point prediction, where the artificial grouping mechanism improves flexibility without altering the global regression coefficients. In the high‐dimensional breast cancer application, VarGuid is used primarily as a sparse predictive and variable‐selection procedure rather than as a formal mechanism‐discovery model for the conditional variance. Thus, the empirical results should be interpreted according to the component being used: Coefficient interpretation for the global linear mean–variance model and RMSE‐based prediction assessment for the grouped extension.

Although the grouped term has a clear statistical interpretation as a local intercept correction, the artificial grouping extension introduces interpretability challenges at the predictor level. In particular, it is not clear how much each predictor contributes to prediction through its influence on artificial group formation. Developing methods to quantify the contribution of individual predictors to the artificial grouping structure is a natural direction for future research.

More broadly, formal inference for the penalized joint estimator, such as asymptotic normality and valid post‐selection standard errors for $\left(\hat{\beta },\hat{\gamma }\right)$, poses additional challenges due to joint penalization and high dimensionality. Formal uncertainty quantification for the grouped extension, including propagation of first‐stage uncertainty from $\left(\hat{\beta },\hat{\gamma }\right)$ into the artificial grouping step, is also outside the scope of the present paper. A fully iterative scheme that re‐estimates $\left(\hat{\beta },\hat{\gamma }\right)$ jointly with the adaptive groups is an interesting direction for future research, but it would amount to a different semiparametric model and would require new identifiability, optimization, and post‐selection inference analysis. Extending variance‐guided regression to incorporate debiased inference, post‐selection inference, or prediction intervals that propagate uncertainty from both estimation stages represents a promising avenue for future development.

## Funding

This work was supported by the National Institute of General Medical Sciences of the National Institutes of Health (Grant No. R35 GM139659); the National Heart, Lung, and Blood Institute of the National Institutes of Health (Grant No. R01 HL164405); and the Medical Research Council (Grant No. MR/P008984/1) under a Global Alliance for Chronic Disease call, and the 2023 Relief Funding Award from the Office of the Vice Provost for Research and Scholarship and the Office of Faculty Affairs, University of Miami.

## Conflicts of Interest

The authors declare no conflicts of interest.

## Supporting information




**Data S1.** Supporting Information.

## Data Availability

We used public data available at: https://github.com/ccchang0111/PAM50. Our code is publicly available as the R‐package varGuid, available on CRAN at https://cloud.r‐project.org/web/packages/varGuid, and at the repository https://github.com/luminwin/varGuid.

## ENDNOTES

^1^
When $\lambda_{\beta }=0$, we use ordinary least squares for the update, and the standard errors of $\hat{\beta }$ are obtained from the weighted least squares estimator with the weights from the final iteration.
