# Supplementary material for: Variance‐Guided Regression for Heteroscedastic Data With a Grouping‐Based Extension for Nonlinear Prediction
Source: Stat Med. 2026 Jun 7;45(13-14):e70632. doi: 10.1002/sim.70632 (PMC13242984; doi:10.1002/sim.70632)
Supplement: Supplementary file 1 — Data S1. Supporting Information. [file SIM-45-0-s001.pdf]

Supplementary Materials for

Variance-Guided Regression for Heteroscedastic Data with a Grouping-Based Extension for Nonlinear Prediction

Appendix A: Theoretical proofs for Section 2

2.1 Assumptions and notation

Let $(Y_i, \mathbf{X}_i)_{i=1}^n$ be i.i.d. with $Y_i \in \mathbb{R}$ and $\mathbf{X}_i \in \mathbb{R}^p$. Let

$$\mathbf{X} = [\mathbf{X}_1^\top, \ldots, \mathbf{X}_n^\top]^\top \in \mathbb{R}^{n \times p}$$

denote the sample design matrix. For population-level statements, let $(Y, \mathbf{X}_0)$ denote a generic observation distributed as $(Y_i, \mathbf{X}_i)$, and let $\boldsymbol{x}$ denote a fixed value of $\mathbf{X}_0$. For $\boldsymbol{\beta}, \boldsymbol{\gamma} \in \mathbb{R}^p$, define

$$\ell(y, \boldsymbol{x}; \boldsymbol{\beta}, \boldsymbol{\gamma}) = \frac{(y - \boldsymbol{x}^\top \boldsymbol{\beta})^2}{2(\boldsymbol{x}^\top \boldsymbol{\gamma})^2} + \log |\boldsymbol{x}^\top \boldsymbol{\gamma}|,$$

well-defined when $|\boldsymbol{x}^\top \boldsymbol{\gamma}| > 0$. Write the *population risk* as

$$R(\boldsymbol{\beta}, \boldsymbol{\gamma}) = \mathbb{E}\{\ell(Y, \mathbf{X}_0; \boldsymbol{\beta}, \boldsymbol{\gamma})\}$$

and the *empirical risk* as

$$R_n(\boldsymbol{\beta}, \boldsymbol{\gamma}) = \frac{1}{n} \sum_{i=1}^n \ell(Y_i, \mathbf{X}_i; \boldsymbol{\beta}, \boldsymbol{\gamma}).$$

Our estimator minimizes the penalized empirical risk

$$\widehat{\boldsymbol{\beta}}, \widehat{\boldsymbol{\gamma}} \in \arg\min_{\boldsymbol{\beta}, \boldsymbol{\gamma}} \; \mathcal{Q}_n(\boldsymbol{\beta}, \boldsymbol{\gamma}) := R_n(\boldsymbol{\beta}, \boldsymbol{\gamma}) + \lambda_\beta \|\boldsymbol{\beta}\|_1 + \lambda_\gamma \|\boldsymbol{\gamma}\|_1.$$

Assumption 1 (Regularity)**.**

(i) *(Finite moments)* $\mathbb{E}\|\mathbf{X}\|_2^2 < \infty$ *and* $\mathbb{E}Y^2 < \infty$.

(ii) *(Innovation moments) The standardized innovation* ε *in the data-generating mechanism satisfies* $\mathbb{E}(\varepsilon) = 0$, $\operatorname{Var}(\varepsilon) = 1$, *and* $\mathbb{E}(\varepsilon^4) < \infty$.

(iii) *(Away from zero variance index) There exists* $c_\eta > 0$ *such that* $|\mathbf{X}^\top \boldsymbol{\gamma}| \geq c_\eta$ *almost surely for all* $\boldsymbol{\gamma}$ *under consideration;*[1]

(iv) *(Restricted strong convexity / eigenvalue) There exist constants* $\kappa > 0$ *and a tolerance* $\tau_n \geq 0$ *such that for all pairs* $(\boldsymbol{\beta}, \boldsymbol{\gamma})$ *and for all perturbations* $(\Delta_\beta, \Delta_\gamma)$ *in the cone*

$$\mathcal{C}(S_\beta, S_\gamma) := \Big\{(\Delta_\beta, \Delta_\gamma) : \|\Delta_{\beta, S_\beta^c}\|_1 \leq 3\|\Delta_{\beta, S_\beta}\|_1, \; \|\Delta_{\gamma, S_\gamma^c}\|_1 \leq 3\|\Delta_{\gamma, S_\gamma}\|_1\Big\},$$

the population risk satisfies the RSC inequality

$$R(\boldsymbol{\beta} + \Delta_\beta, \boldsymbol{\gamma} + \Delta_\gamma) - R(\boldsymbol{\beta}, \boldsymbol{\gamma}) \;\geq\; \frac{\kappa}{2}\Big(\|\Delta_\beta\|_2^2 + \|\Delta_\gamma\|_2^2\Big) - \tau_n\Big(\|\Delta_\beta\|_1 + \|\Delta_\gamma\|_1\Big)^2.$$

Here S_β, S_γ *are index sets (e.g. supports of oracle parameters) with sizes* $s_\beta = |S_\beta|$, $s_\gamma = |S_\gamma|$.

[1] As is standard in heteroscedastic modeling, this restriction avoids divisions by zero and enforces identifiability of the scale index on the parameter set of interest; practically one uses a small stabilizer ϵ in the algorithm.

2.2 Population dominance (risk nesting)

Proposition 2.1 (Population quasi-risk dominance)**.** *Let* $(Y, \mathbf{X}_0)$ *denote a generic observation distributed as* $(Y_i, \mathbf{X}_i)$*, and let* $\boldsymbol{x}$ *denote a fixed value of* $\mathbf{X}_0$*. Let*

$$(\boldsymbol{\beta}^\star, \boldsymbol{\gamma}^\star) \in \arg\min_{\boldsymbol{\beta},\boldsymbol{\gamma}} R(\boldsymbol{\beta},\boldsymbol{\gamma}), \qquad (\boldsymbol{\beta}^{\mathrm{hom}}, \boldsymbol{\gamma}^{\mathrm{hom}}) \in \arg \min_{\boldsymbol{\beta},\boldsymbol{\gamma}:\, \boldsymbol{x}^\top\boldsymbol{\gamma}\equiv c>0} R(\boldsymbol{\beta},\boldsymbol{\gamma}).$$

Under Assumption 1, we have

$$R(\boldsymbol{\beta}^\star, \boldsymbol{\gamma}^\star) \;\le\; R(\boldsymbol{\beta}^{\mathrm{hom}}, \boldsymbol{\gamma}^{\mathrm{hom}}).$$

Moreover, the inequality is strict whenever no homoscedastic pair attains the full-class minimum. In particular, this occurs when the full-class minimizer is unique and the covariate-specific optimal scale

$$\eta^\star(\boldsymbol{x}) \in \arg\min_{u>0} \mathbb{E}\left[\frac{(Y-\boldsymbol{x}^\top\boldsymbol{\beta}^\star)^2}{2u^2} + \log u \,\middle|\, \mathbf{X}_0 = \boldsymbol{x}\right]$$

is nonconstant on a set of positive measure and is attained by the admissible variance-index class.

Proof. Let

$$\mathcal{H}_{\mathrm{hom}} = \{(\boldsymbol{\beta},\boldsymbol{\gamma}) : \boldsymbol{x}^\top\boldsymbol{\gamma} \equiv c > 0\}$$

denote the homoscedastic class. Then $\mathcal{H}_{\mathrm{hom}} \subset \{(\boldsymbol{\beta},\boldsymbol{\gamma}) : |\boldsymbol{x}^\top\boldsymbol{\gamma}| > 0\}$, so by nesting

$$\inf_{\boldsymbol{\beta},\boldsymbol{\gamma}} R(\boldsymbol{\beta},\boldsymbol{\gamma}) \;\le\; \inf_{(\boldsymbol{\beta},\boldsymbol{\gamma})\in\mathcal{H}_{\mathrm{hom}}} R(\boldsymbol{\beta},\boldsymbol{\gamma}),$$

which yields $R(\boldsymbol{\beta}^\star, \boldsymbol{\gamma}^\star) \le R(\boldsymbol{\beta}^{\mathrm{hom}}, \boldsymbol{\gamma}^{\mathrm{hom}})$.

For strictness, suppose equality holds. Then a homoscedastic minimizer also attains the minimum over the full heteroscedastic class. Therefore, strict inequality holds whenever no homoscedastic pair attains the full-class minimum.

To connect this condition to the covariate-specific scale, fix $\boldsymbol{\beta} = \boldsymbol{\beta}^\star$ and consider, for each fixed $\boldsymbol{x}$,

$$\varphi_{\boldsymbol{x}}(u) = \mathbb{E}\left[\frac{(Y-\boldsymbol{x}^\top\boldsymbol{\beta}^\star)^2}{2u^2} + \log u \,\middle|\, \mathbf{X}_0 = \boldsymbol{x}\right], \qquad u > 0.$$

Write

$$a(\boldsymbol{x}) = \mathbb{E}\big[(Y-\boldsymbol{x}^\top\boldsymbol{\beta}^\star)^2 \,\big|\, \mathbf{X}_0 = \boldsymbol{x}\big].$$

Then

$$\varphi'_{\boldsymbol{x}}(u) = -\frac{a(\boldsymbol{x})}{u^3} + \frac{1}{u}, \qquad \varphi''_{\boldsymbol{x}}(u) = \frac{3a(\boldsymbol{x})}{u^4} - \frac{1}{u^2}.$$

Thus $\varphi_{\boldsymbol{x}}(u)$ need not be globally strictly convex, but it has a unique minimizer at

$$\eta^\star(\boldsymbol{x}) = \sqrt{a(\boldsymbol{x})},$$

under the nondegenerate variance condition. If this optimal scale is nonconstant on a set of positive measure and is attained by the admissible heteroscedastic class, then no constant homoscedastic scale can attain the same pointwise optimum. In particular, if the full-class minimizer is unique, equality with the homoscedastic minimum is impossible. Hence the inequality is strict. □

Proposition 2.2 (Nesting for penalized population objective)**.** *Fix any nonnegative penalty* $P(\boldsymbol{\beta},\boldsymbol{\gamma}) \ge 0$ *(e.g.,* $P(\boldsymbol{\beta},\boldsymbol{\gamma}) = \lambda_\beta\|\boldsymbol{\beta}\|_1 + \lambda_\gamma\|\boldsymbol{\gamma}\|_1$*) that does not depend on the model class. Define* $J(\boldsymbol{\beta},\boldsymbol{\gamma}) = R(\boldsymbol{\beta},\boldsymbol{\gamma}) + P(\boldsymbol{\beta},\boldsymbol{\gamma})$*. Let* $(\boldsymbol{\beta}^\dagger, \boldsymbol{\gamma}^\dagger) \in \arg\min_{\boldsymbol{\beta},\boldsymbol{\gamma}} J(\boldsymbol{\beta},\boldsymbol{\gamma})$ *and* $(\boldsymbol{\beta}^{\dagger,\mathrm{hom}}, \boldsymbol{\gamma}^{\dagger,\mathrm{hom}}) \in \arg\min_{\boldsymbol{\beta},\boldsymbol{\gamma}:\, x^\top\boldsymbol{\gamma}\equiv c>0} J(\boldsymbol{\beta},\boldsymbol{\gamma})$*. Then* $J(\boldsymbol{\beta}^\dagger, \boldsymbol{\gamma}^\dagger) \le J(\boldsymbol{\beta}^{\dagger,\mathrm{hom}}, \boldsymbol{\gamma}^{\dagger,\mathrm{hom}})$*.*

Proof. The feasible homoscedastic set is a subset of the full parameter space; taking the infimum of the same objective J over a superset cannot be larger. □

2.3 High-dimensional oracle inequality

We now give a finite-sample bound comparing the predictive risk of the empirical penalized estimator to that of the best sparse oracle pair. Let

$$(\boldsymbol{\beta}^\star, \boldsymbol{\gamma}^\star) \in \arg \min_{\boldsymbol{\beta},\boldsymbol{\gamma}:\ \|\boldsymbol{\beta}\|_0 \le s_\beta,\ \|\boldsymbol{\gamma}\|_0 \le s_\gamma} R(\boldsymbol{\beta}, \boldsymbol{\gamma}), \quad S_\beta = \operatorname{supp}(\boldsymbol{\beta}^\star),\ S_\gamma = \operatorname{supp}(\boldsymbol{\gamma}^\star).$$

Theorem 2.3 (Oracle inequality)**.** *Suppose Assumption 1 holds, and choose tuning parameters*

$$\lambda_\beta \ \ge\ 2\left\|\nabla_{\boldsymbol{\beta}} R_n(\boldsymbol{\beta}^\star, \boldsymbol{\gamma}^\star) - \nabla_{\boldsymbol{\beta}} R(\boldsymbol{\beta}^\star, \boldsymbol{\gamma}^\star)\right\|_\infty, \qquad \lambda_\gamma \ \ge\ 2\left\|\nabla_{\boldsymbol{\gamma}} R_n(\boldsymbol{\beta}^\star, \boldsymbol{\gamma}^\star) - \nabla_{\boldsymbol{\gamma}} R(\boldsymbol{\beta}^\star, \boldsymbol{\gamma}^\star)\right\|_\infty.$$

Then any global minimizer $(\widehat{\boldsymbol{\beta}}, \widehat{\boldsymbol{\gamma}})$ of $\mathcal{Q}_n$ satisfies, with probability at least $1-\delta$,

$$R(\widehat{\boldsymbol{\beta}}, \widehat{\boldsymbol{\gamma}}) - R(\boldsymbol{\beta}^\star, \boldsymbol{\gamma}^\star) \ \le\ \frac{9}{\kappa}\left(\lambda_\beta^2 s_\beta + \lambda_\gamma^2 s_\gamma\right) \ +\ \frac{4\tau_n}{\kappa}\left(\lambda_\beta s_\beta + \lambda_\gamma s_\gamma\right),$$

where κ and τ_n are the RSC constants from Assumption 1(iv). In particular, if $\tau_n = 0$ and $\lambda_\beta, \lambda_\gamma \asymp \sqrt{(\log p)/n}$ (e.g. via standard concentration bounds), then

$$R(\widehat{\boldsymbol{\beta}}, \widehat{\boldsymbol{\gamma}}) - R(\boldsymbol{\beta}^\star, \boldsymbol{\gamma}^\star) \ \lesssim\ (s_\beta + s_\gamma)\,\frac{\log p}{n}.$$

Proof. Step 1: Basic inequality and decomposability. By optimality of $(\widehat{\boldsymbol{\beta}}, \widehat{\boldsymbol{\gamma}})$ and feasibility of $(\boldsymbol{\beta}^\star, \boldsymbol{\gamma}^\star)$,

$$R_n(\widehat{\boldsymbol{\beta}}, \widehat{\boldsymbol{\gamma}}) + \lambda_\beta\|\widehat{\boldsymbol{\beta}}\|_1 + \lambda_\gamma\|\widehat{\boldsymbol{\gamma}}\|_1 \ \le\ R_n(\boldsymbol{\beta}^\star, \boldsymbol{\gamma}^\star) + \lambda_\beta\|\boldsymbol{\beta}^\star\|_1 + \lambda_\gamma\|\boldsymbol{\gamma}^\star\|_1.$$

Add and subtract $R(\cdot)$ and rearrange to get

$$\underbrace{R(\widehat{\boldsymbol{\beta}}, \widehat{\boldsymbol{\gamma}}) - R(\boldsymbol{\beta}^\star, \boldsymbol{\gamma}^\star)}_{:=\Delta_R} \ \le\ \underbrace{\left[R(\boldsymbol{\beta}^\star, \boldsymbol{\gamma}^\star) - R_n(\boldsymbol{\beta}^\star, \boldsymbol{\gamma}^\star)\right] - \left[R(\widehat{\boldsymbol{\beta}}, \widehat{\boldsymbol{\gamma}}) - R_n(\widehat{\boldsymbol{\beta}}, \widehat{\boldsymbol{\gamma}})\right]}_{:=\mathcal{E}_n} + \lambda_\beta\left(\|\boldsymbol{\beta}^\star\|_1 - \|\widehat{\boldsymbol{\beta}}\|_1\right) + \lambda_\gamma\left(\|\boldsymbol{\gamma}^\star\|_1 - \|\widehat{\boldsymbol{\gamma}}\|_1\right).$$

By convexity of the ℓ_1 norm and decomposability,

$$\|\boldsymbol{\beta}^\star\|_1 - \|\widehat{\boldsymbol{\beta}}\|_1 \ \le\ \|\Delta_{\beta,S_\beta}\|_1 - \|\Delta_{\beta,S_\beta^c}\|_1, \qquad \|\boldsymbol{\gamma}^\star\|_1 - \|\widehat{\boldsymbol{\gamma}}\|_1 \ \le\ \|\Delta_{\gamma,S_\gamma}\|_1 - \|\Delta_{\gamma,S_\gamma^c}\|_1,$$

where $\Delta_\beta = \widehat{\boldsymbol{\beta}} - \boldsymbol{\beta}^\star$ and $\Delta_\gamma = \widehat{\boldsymbol{\gamma}} - \boldsymbol{\gamma}^\star$.

Step 2: Bounding the empirical process term. A first-order mean value expansion of $R_n - R$ along the segment joining $(\boldsymbol{\beta}^\star, \boldsymbol{\gamma}^\star)$ and $(\widehat{\boldsymbol{\beta}}, \widehat{\boldsymbol{\gamma}})$ yields

$$\mathcal{E}_n \ \le\ \left\langle \nabla_{\boldsymbol{\beta}} R_n(\boldsymbol{\beta}^\star, \boldsymbol{\gamma}^\star) - \nabla_{\boldsymbol{\beta}} R(\boldsymbol{\beta}^\star, \boldsymbol{\gamma}^\star),\ \Delta_\beta\right\rangle + \left\langle \nabla_{\boldsymbol{\gamma}} R_n(\boldsymbol{\beta}^\star, \boldsymbol{\gamma}^\star) - \nabla_{\boldsymbol{\gamma}} R(\boldsymbol{\beta}^\star, \boldsymbol{\gamma}^\star),\ \Delta_\gamma\right\rangle.$$

By Hölder's inequality and the choice of $\lambda_\beta, \lambda_\gamma$,

$$\mathcal{E}_n \ \le\ \frac{\lambda_\beta}{2}\|\Delta_\beta\|_1 + \frac{\lambda_\gamma}{2}\|\Delta_\gamma\|_1.$$

Step 3: Cone condition. Plugging the bounds from Steps 1–2 into the basic inequality and regrouping gives

$$\Delta_R \ \le\ \frac{\lambda_\beta}{2}\|\Delta_\beta\|_1 + \frac{\lambda_\gamma}{2}\|\Delta_\gamma\|_1 + \lambda_\beta\left(\|\Delta_{\beta,S_\beta}\|_1 - \|\Delta_{\beta,S_\beta^c}\|_1\right) + \lambda_\gamma\left(\|\Delta_{\gamma,S_\gamma}\|_1 - \|\Delta_{\gamma,S_\gamma^c}\|_1\right).$$

Canceling terms, one obtains the cone relations

$$\|\Delta_{\beta,S_\beta^c}\|_1 \ \le\ 3\|\Delta_{\beta,S_\beta}\|_1, \qquad \|\Delta_{\gamma,S_\gamma^c}\|_1 \ \le\ 3\|\Delta_{\gamma,S_\gamma}\|_1,$$

so $(\Delta_\beta, \Delta_\gamma) \in \mathcal{C}(S_\beta, S_\gamma)$.

Step 4: RSC lower bound and completing the square. By Assumption 1(iv) (RSC) applied at $(\boldsymbol{\beta}^\star, \boldsymbol{\gamma}^\star)$ in the direction $(\Delta_\beta, \Delta_\gamma) \in \mathcal{C}$,

$$\Delta_R \ \ge\ \frac{\kappa}{2}\left(\|\Delta_\beta\|_2^2 + \|\Delta_\gamma\|_2^2\right) - \tau_n\left(\|\Delta_\beta\|_1 + \|\Delta_\gamma\|_1\right)^2.$$

Combine the upper and lower bounds on Δ_R and use the cone condition plus $\|\Delta_{\beta,S_\beta}\|_1 \le \sqrt{s_\beta}\|\Delta_\beta\|_2$ (and analogously for γ) to obtain

$$\frac{\kappa}{2}\big(\|\Delta_\beta\|_2^2 + \|\Delta_\gamma\|_2^2\big) \;\le\; \frac{3\lambda_\beta}{2}\|\Delta_{\beta,S_\beta}\|_1 + \frac{3\lambda_\gamma}{2}\|\Delta_{\gamma,S_\gamma}\|_1 + \tau_n\Big(4\|\Delta_{\beta,S_\beta}\|_1^2 + 4\|\Delta_{\gamma,S_\gamma}\|_1^2\Big).$$

Using $\|\Delta_{\beta,S_\beta}\|_1 \le \sqrt{s_\beta}\|\Delta_\beta\|_2$ and Young's inequality yields

$$\|\Delta_\beta\|_2^2 + \|\Delta_\gamma\|_2^2 \;\le\; \frac{9}{\kappa^2}\big(\lambda_\beta^2 s_\beta + \lambda_\gamma^2 s_\gamma\big) + \frac{8\tau_n}{\kappa}\big(\lambda_\beta s_\beta + \lambda_\gamma s_\gamma\big).$$

Finally, apply the RSC lower bound once more to translate the squared ℓ_2 bound into an excess-risk bound:

$$\Delta_R \;\le\; \frac{\kappa}{2}\big(\|\Delta_\beta\|_2^2 + \|\Delta_\gamma\|_2^2\big) \;\le\; \frac{9}{\kappa}\big(\lambda_\beta^2 s_\beta + \lambda_\gamma^2 s_\gamma\big) + \frac{4\tau_n}{\kappa}\big(\lambda_\beta s_\beta + \lambda_\gamma s_\gamma\big),$$

which is the stated inequality. □

Remark (On the choice of $\lambda_\beta, \lambda_\gamma$). Under Assumption 1(i)–(iii) and standard moment/concentration bounds for empirical processes (the gradients are averages of sub-exponential terms because $(X^\top \boldsymbol{\gamma})^{-k}$ is bounded by c_η^{-k}), one may take $\lambda_\beta, \lambda_\gamma \asymp \sqrt{(\log p)/n}$ so that $\|\nabla R_n(\boldsymbol{\beta}^\star, \boldsymbol{\gamma}^\star) - \nabla R(\boldsymbol{\beta}^\star, \boldsymbol{\gamma}^\star)\|_\infty = O_p\big(\sqrt{(\log p)/n}\big)$.

Appendix B: Simulations for Section 2 with linear effects

As shown in Table S1, we consider five simulation scenarios where Scenario 1 is designed to assess the power of correlation coefficient and Scenario 3 examines the result of regular least square. For each scenario, we generate the features and ε_i from standard normal distribution with 1000 replications for two levels of sample size $n = 20$ and $n = 200$ and two levels of correlation coefficients among features $\rho = 0$ and $\rho = 0.9$. The results of heteroscedasticity testing using the Breusch-Pagan test are presented in Table S1, showing the proportion of significant test statistics ($p \le .05$) among all the 1000 replications. For the high-dimensional scenarios, the tuning parameters λ_β and λ_γ for Algorithm 1 were chosen by grid search with 10-fold cross-validation, and the Lasso was implemented using 10-fold cross-validation in the `glmnet` R package (Friedman et al. 2010).

When evaluating bias, mean squared error (MSE), and confidence interval (CI) coverage for the low-dimensional scenarios in Table S2, we averaged the results across coefficients with true values $\beta_j = 1$ and $\beta_j = 0$. The bias results show that VarGuid generally yields lower bias than OLS. The MSE results further demonstrate that VarGuid consistently outperforms OLS by producing smaller errors, most notably in settings with small samples and highly correlated predictors. In the homoscedastic setting (Scenario 3), VarGuid performs comparably to OLS, indicating that it does not overfit when variance heterogeneity is absent. These findings suggest that VarGuid provides more reliable parameter estimates by accounting for variance structure in the data. Table S2 also shows that heteroskedasticity-robust standard errors tend to be conservative relative to those from WLS; accordingly, we rely on WLS-based standard errors in the low-dimensional real-data application. Table S3 presents results in terms of root mean squared error (RMSE) for predicted outcomes and area under the precision–recall curve (AUC-PR) for variable selection of $\boldsymbol{\beta}$. Scenarios 6–8 extend Scenario 5 by systematically increasing the dimensionality to $p = 20, 100, 200$, respectively. As the number of predictors grows, the baseline AUC-PR values under random selection decrease substantially (0.25, 0.05, and 0.025). The results demonstrate that incorporating VarGuid alongside Lasso consistently improves both predictive accuracy and variable selection performance.

Table S1: Design of simulation scenarios.

#	$\boldsymbol{\beta}$	$\boldsymbol{\gamma}$	p§	Significant B-P test proportion†			
				n=20		n=200	
				$\rho=0$	$\rho=.9$	$\rho=0$	$\rho=.9$
1	$\beta_1 = 1$	$\gamma_1 = 1$	1	0.669	-	1	-
2	$(\beta_1, \beta_2, \beta_3, \beta_4, \beta_5)^T = (1,1,1,1,1)^T$	$(\gamma_1, \gamma_2, \gamma_3, \gamma_4, \gamma_5)^T = (1,1,1,1,1)^T$	10	0.156	0.166	0.94	0.748
3	$(\beta_1, \beta_2, \beta_3, \beta_4, \beta_5)^T = (1,1,1,1,1)^T$	$(\mathbf{x}_i\boldsymbol{\gamma})\varepsilon_i = \varepsilon_i \ \forall i$	10	0.032	0.034	0.05	0.048
4	$(\beta_1, \beta_2, \beta_3, \beta_4, \beta_5)^T = (1,1,1,1,1)^T$	$(\gamma_6, \gamma_7, \gamma_8, \gamma_9, \gamma_{10})^T = (1,1,1,1,1)^T$	10	0.078	0.154	0.323	0.726
5	$(\beta_1, \beta_2, \beta_3, \beta_4, \beta_5)^T = (1,1,1,1,1)^T$	$(\gamma_1, \gamma_2, \gamma_3, \gamma_4, \gamma_5, \gamma_6)^T = (0,1,2,3,4,5)^T$	15	0.073	0.065	0.371	0.518

§ All the other β_j and γ_j equal to zero if not specified.
† B-P is short for Breusch Pagan test with a significant level of .05.

Appendix C: Simulations for Section 3 with nonlinear and interaction effects

Figure S1 examines the behavior of the artificial grouping algorithm when the true outcome is linear, $Y_i = -X_i + \varepsilon_i$. Panel leashf2A shows that adding an artificial grouping term does not improve prediction beyond the linear model, with the optimal tuning parameter ζ driving the solution toward a single group. Panel S1B displays the corresponding fusion path for the subgroup centers: as ζ increases, centers merge rapidly and ultimately collapse into one cluster. Panel S1C presents the cross-validated RMSE curve used to select ζ, which correctly favors large values and returns a single-group structure. Together, these results confirm that the grouping mechanism does not spuriously introduce nonlinear adjustments when the true mean function is linear.

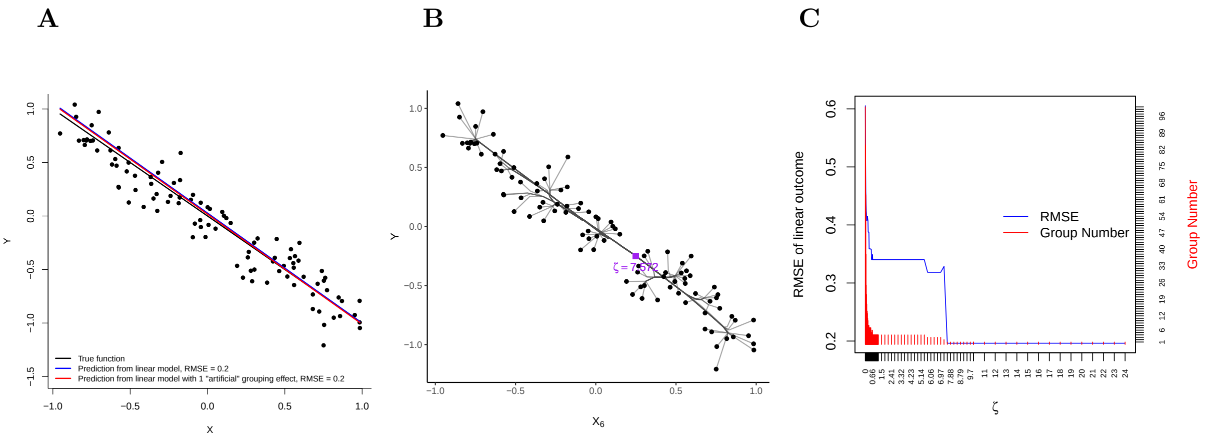

Figure S1: **A**: Simulation with a linear outcome $Y_i = -X_i + \varepsilon_i$ used to assess whether the artificial grouping procedure can overfit a linear model. The other settings of this simulation are the same as in Figure 2A–C. **B**: Path of subgroup centers as ζ increases; larger ζ yields more subgroup fusion. As in the nonlinear case of Figure 2B, the fusion path is driven primarily by the covariate pattern rather than by residual structure. As ζ increases from 0 to 7.572, the centers merge progressively, reducing from n distinct groups to a single group. **C**: Test-set RMSE from Panel **A** as a function of ζ, used to select the tuning parameter and the corresponding number of groups. For this linear outcome, the RMSE curve favors large values of ζ, returning a single-group solution, which coincides with the simple linear regression shown by the red line in Panel **A**.

For nonlinear settings, the simulations used to test VarGuid were of the form $Y = \psi(\mathbf{x}) + \varepsilon$ or $Y = \psi(\mathbf{x}, \varepsilon)$ and are listed below:

1. cobra2: $\psi(\mathbf{x}) = x^{(1)}x^{(2)} + (x^{(3)})^2 - x^{(4)}x^{(7)} + x^{(8)}x^{(10)} - (x^{(6)})^2$, $X^{(j)} \sim U(-1,1)$, $\varepsilon \sim N(0, 0.1^2)$.
2. cobra8: $\psi(\mathbf{x}, \varepsilon) = I\{x^{(1)} + (x^{(4)})^3 + x^{(9)} + \sin(x^{(2)}x^{(8)}) + \varepsilon > 0.38\}$, $X^{(j)} \sim U(-.25, 1)$, $\varepsilon \sim N(0, 0.1^2)$.

Table S2: Simulation results: bias, MSE, and CI coverage in estimating $\boldsymbol{\beta}$

Bias

	$\beta_j = 1$								$\beta_j = 0$							
	OLS[†]				VarGuid				OLS				VarGuid			
	$n = 20$		$n = 200$		$n = 20$		$n = 200$		$n = 20$		$n = 200$		$n = 20$		$n = 200$	
# \ ρ	0	.9	0	.9	0	.9	0	.9	0	.9	0	.9	0	.9	0	.9
1	-0.017		-0.001		-0.010		-0.000									
2	0.001	-0.110	0.005	-0.001	0.001	-0.070	0.004	-0.001	-0.005	0.112	0.002	0.001	-0.001	0.069	0.001	0.001
3	-0.001	0.000	-0.001	0.000	-0.001	0.000	0.001	0.000	0.000	0.001	0.000	0.001	0.000	-0.001	0.000	0.001
4	-0.024	-0.060	0.001	0.012	-0.014	-0.018	0.001	0.010	-0.002	0.059	0.002	-0.013	-0.001	0.019	0.002	-0.010
5	0.028	-0.447	0.010	-0.003	0.004	-0.126	0.001	-0.001	-0.014	0.024	0.005	0.025	0.012	0.013	0.004	0.023

MSE

	$\beta_j = 1$								$\beta_j = 0$							
	OLS				VarGuid				OLS				VarGuid			
	$n = 20$		$n = 200$		$n = 20$		$n = 200$		$n = 20$		$n = 200$		$n = 20$		$n = 200$	
# \ ρ	0	.9	0	.9	0	.9	0	.9	0	.9	0	.9	0	.9	0	[illegible]
1	0.195		0.019		0.179	0.011										
2	0.818	23.546	0.041	1.165	0.740	21.406	0.040	1.126	0.735	23.357	0.032	1.131	0.662	21.215	0.030	[illegible]
3	0.127	1.093	0.005	0.051	0.115	0.992	0.005	0.051	0.126	1.112	0.005	0.049	0.115	1.000	0.005	[illegible]
4	0.715	24.850	0.032	1.107	0.631	22.263	0.030	1.070	0.811	24.814	0.042	1.206	0.709	22.591	0.040	[illegible]
5	17.690	619.627	0.307	10.731	13.536	467.261	0.300	10.552	18.697	585.485	0.363	10.775	13.973	449.772	0.359	[illegible]

CI coverage ($\beta_j = 1$)

	WLS				Sandwich (from $\hat{\boldsymbol{\Sigma}}_{\mathbf{0}}$)				Jackknife (from $\hat{\boldsymbol{\Sigma}}_{\mathbf{1}}$)			
	$n = 20$		$n = 200$		$n = 20$		$n = 200$		$n = 20$		$n = 200$	
# \ ρ	0	0.9	0	0.9	0	0.9	0	0.9	0	0.9	0	0.9
1	0.951		0.954		0.943		0.944		0.887		0.943	
2	0.933	0.952	0.947	0.948	0.993	0.994	0.956	0.953	0.774	0.817	0.940	0.935
3	0.953	0.954	0.955	0.940	0.991	0.991	0.958	0.947	0.809	0.801	0.941	0.926
4	0.957	0.952	0.953	0.955	0.994	0.993	0.957	0.960	0.818	0.811	0.939	0.943
5	0.954	0.962	0.955	0.948	0.999	0.999	0.962	0.959	0.707	0.695	0.941	0.934

CI coverage ($\beta_j \neq 0$)

	WLS				Sandwich (from $\hat{\boldsymbol{\Sigma}}_{\mathbf{0}}$)				Jackknife (from $\hat{\boldsymbol{\Sigma}}_{\mathbf{1}}$)			
	$n = 20$		$n = 200$		$n = 20$		$n = 200$		$n = 20$		$n = 200$	
# \ ρ	0	0.9	0	0.9	0	0.9	0	0.9	0	0.9	0	0.9
2	0.953	0.959	0.954	0.956	0.995	0.995	0.958	0.960	0.808	0.814	0.943	0.944
3	0.952	0.951	0.946	0.943	0.991	0.988	0.949	0.945	0.803	0.795	0.933	0.930
4	0.940	0.948	0.954	0.946	0.993	0.992	0.953	0.951	0.783	0.805	0.933	0.936
5	0.953	0.963	0.946	0.952	0.999	0.999	0.957	0.961	0.686	0.706	0.932	0.937

[†] The point estimators from White's heteroskedasticity-consistent estimators and other variance-robust methods are identical to OLS; only the standard errors differ. Therefore, for bias and MSE comparisons, we report OLS as the representative baseline.

Table S3: Simulation results: RMSE for predicted outcomes and AUC-PR for variable selection of $\boldsymbol{\beta}$ across Scenarios 5–8.

RMSE	Lasso				VarGuid			
	$n = 20$		$n = 200$		$n = 20$		$n = 200$	
# \ ρ	0	.9	0	.9	0	.9	0	.9
5	8.258	16.419	8.133	16.022	7.426	14.310	6.970	13.970
6	9.409	17.238	8.219	16.136	7.487	15.311	7.233	14.238
7	9.992	19.131	8.516	16.925	7.555	16.981	7.459	15.782
8	11.072	20.614	10677	20.019	7.928	18.395	7.743	16.003

AUC-PR	Lasso				VarGuid			
	$n = 20$		$n = 200$		$n = 20$		$n = 200$	
# \ ρ	0	.9	0	.9	0	.9	0	.9
5	0.645	0.426	0.768	0.708	0.794	0.718	0.908	0.888
6	0.575	0.309	0.580	0.578	0.616	0.560	0.611	0.648
7	0.160	0.089	0.128	0.110	0.263	0.112	0.584	0.188
8	0.102	0.053	0.080	0.058	0.128	0.076	0.110	0.096

3. friedman1: $\psi(\mathbf{x}) = 10\sin(\pi x^{(1)}x^{(2)}) + 20(x^{(3)} - 0.5)^2 + 10x^{(4)} + 5x^{(5)}$, $X^{(j)} \sim U(0,1)$, $\varepsilon \sim N(0,1)$.
4. friedman3: $\psi(\mathbf{x}) = \arctan\left[\frac{x^{(2)}x^{(3)} - 1/(x^{(2)}x^{(4)})}{x^{(1)}}\right]$, $X^{(1)} \sim U(0,100)$, $X^{(2)} \sim U(40\pi, 560\pi)$, $X^{(3)},\ldots,X^{(p)} \sim U(0,1)$, $\varepsilon \sim N(0,1)$.
5. inx1: $\psi(\mathbf{x}) = x^{(1)}(x^{(2)})^2\sqrt{|x^{(3)}|} + \lfloor x^{(4)} - x^{(5)}x^{(6)}\rfloor$, $X^{(j)} \sim U(-1,1)$, $\varepsilon \sim N(0, 0.1^2)$.
6. inx2: $\psi(\mathbf{x}) = x^{(3)}(x^{(1)}+1)^{|x^{(2)}|} - \sqrt{\frac{(x^{(5)})^2}{|x^{(4)}| + |x^{(5)}| + |x^{(6)}|}}$, $X^{(j)} \sim U(-1,1)$, $\varepsilon \sim N(0, 0.1^2)$.
7. inx3: $\psi(\mathbf{x}) = \cos(x^{(1)} - x^{(2)}) + \arcsin(x^{(1)}x^{(3)}) - \arctan(x^{(2)} - (x^{(3)})^2)$, $X^{(j)} \sim U(-1,1)$, $\varepsilon \sim N(0, 0.1^2)$.
8. lmi2: $\psi(\mathbf{x}) = 3(\sum_{j=1}^{15} x^{(j)})^2$, $X^{(j)} \sim N(0,1)$, $\varepsilon \sim N(0, 15^2)$.
9. sup: $\psi(\mathbf{x}) = 10x^{(1)}x^{(2)} + .25\frac{1}{x^{(3)}x^{(4)} + 10x^{(5)}x^{(6)}}$, $X^{(j)} \sim U(0.05, 1)$, $\varepsilon \sim N(0, 0.5^2)$.
10. sup2: $\psi(\mathbf{x}) = \pi^{x^{(1)}x^{(2)}}\sqrt{2x^{(3)}} - \arcsin(x^{(4)}) + \log(x^{(3)} + x^{(5)}) - \frac{x^{(9)}}{x^{(10)}}\sqrt{\frac{x^{(7)}}{x^{(8)}}} - x^{(2)}x^{(7)}$, $X^{(j)} \sim U(0.5, 1)$, $\varepsilon \sim N(0, 0.5^2)$.

Simulations *cobra* follow Biau et al. (2016), and simulations *friedman* follow Friedman (1991). In the independent–feature scenario, the predictors $\mathbf{X}$ were sampled independently as described earlier. In the correlated–feature scenario, all predictors retained their original marginal distributions but were transformed using a copula to induce pairwise correlation $\rho = 0.9$. This procedure was used for all simulations except *lm* and *lmi2*, in which the 15 signal predictors $\mathbf{X}_1, \ldots, \mathbf{X}_{15}$ were correlated within blocks of size five (blocks 1–5, 6–10, and 11–15).

All methods were implemented within a unified simulation framework. For each Monte Carlo replication, the data were split into 80% training and 20% testing sets and this process was repeated 100 times for each scenario. VarGuid was fit with $\lambda_\beta = \lambda_\gamma = 0$ in the low-dimensional settings. Comparative models were implemented through standard R libraries with widely accepted hyperparameter defaults. Penalized regression was fit using `glmnet` (Friedman et al. 2010) with 10-fold cross-validation. Random Forests were trained using `ranger` (Wright & Ziegler 2017) with 500 trees. BART models were estimated using `dbarts` (Dorie 2025) with default priors and MCMC settings. Gradient boosting was fit using `gbm3` (Friedman 2001, Ridgeway & Developers 2024) with 5-fold cross-validation for tuning. XGBoost and LightGBM were trained via their respective R interfaces with RMSE-based objectives and early stopping. When a CatBoost code block

appeared, the model structure followed the LightGBM specification; full CatBoost behavior would require the `catboost` R package (CatBoost Developers 2024). Finally, gradient-boosted generalized additive models were estimated using `mboost` (Hofner et al. 2015, 2014, Hothorn et al. 2010, Buehlmann & Hothorn 2007), with the number of boosting iterations selected by 5-fold cross-validation.

All predictions were evaluated on held-out test sets, and RMSE was used for ranking and critical-difference analysis.

Table S4: RMSE comparison for low dimensional datasets shown in Figure 3(A).

Dataset	VarGuid	flexMix	regmixEM	Random Forest	BART	Gradient Boosting	XGBoost	LightGBM	CatBoost
Concrete	0.537	0.725	0.926	5.433	4.276	4.431	4.016	4.039	4.039
Liver Disorders	0.510	0.652	0.956	4.135	3.544	3.611	3.570	3.746	3.746
Airfoil Self-Nois	3.475	3.254	7.205	3.561	3.535	3.141	2.824	2.996	2.994
Real Estate Valuation	3.465	3.275	6.802	4.526	4.190	4.238	4.176	4.432	4.430
Average Localization Error	3.171	3.003	6.180	3.654	3.382	3.429	3.379	3.589	3.588
Auto MPG	0.720	0.199	0.501	2.904	2.804	2.938	2.969	3.207	3.207
Concrete Slump Test	0.488	0.167	0.422	4.402	1.922	3.050	3.397	3.319	3.319
Yacht Hydrodynamics	1.220	0.596	1.254	3.543	1.759	2.843	1.881	4.372	4.372
Demand Forecasting Orders	0.975	0.474	1.085	4.300	4.905	6.903	3.607	7.474	7.474
Facebook Metrics	0.875	0.420	1.046	4.482	3.344	5.305	3.359	9.761	9.762
cobra2	0.120	0.199	0.501	0.244	0.176	0.178	0.206	0.186	0.186
cobra8	0.188	0.167	0.422	0.191	0.157	0.157	0.170	0.162	0.162
friedman1	0.220	0.596	1.254	0.661	0.640	0.697	0.896	0.773	0.773
friedman3	0.455	0.474	1.085	0.539	0.462	0.456	0.479	0.513	0.513
inx1	0.345	0.420	1.046	0.489	0.427	0.420	0.438	0.470	0.470
inx2	0.382	0.370	0.925	0.440	0.379	0.372	0.389	0.416	0.416
inx3	0.340	0.348	0.927	0.408	0.347	0.340	0.355	0.381	0.381
lmi2	2.595	2.978	7.036	2.935	2.513	2.913	2.866	2.888	2.888
sup	2.771	3.275	6.802	2.700	2.772	2.605	2.741	2.833	2.833
sup2	2.071	3.003	6.180	2.882	2.329	2.398	2.506	2.607	2.607
cobra2*	0.782	0.370	0.927	0.601	0.236	0.352	0.507	0.409	0.409
cobra8*	0.247	0.348	1.027	0.425	0.234	0.281	0.359	0.308	0.308
friedman1*	1.595	2.978	7.836	1.679	0.558	0.548	0.588	0.590	0.590
friedman3 *	0.771	3.532	8.832	0.933	0.815	0.558	0.709	0.618	0.618
inx1*	0.346	3.233	8.022	0.847	0.488	0.529	0.659	0.581	0.581
inx2*	0.537	0.725	0.926	0.759	0.539	0.571	0.584	0.514	0.514
inx3*	0.510	0.652	0.956	0.716	0.404	0.436	0.538	0.475	0.475
lmi2*	3.475	3.254	7.205	3.110	3.617	3.687	3.036	3.333	3.333
sup*	3.065	3.532	8.832	3.135	2.526	2.964	3.002	3.638	3.638
sup2*	2.346	3.233	8.022	2.940	2.963	2.721	2.525	3.337	3.337

† Simulated datasets – cobra2, cobra8, friedman1,friedman3, inx1, inx2, inx3,lmi2, sup and sup2 – used a sample size of $N = 500$ and dimension $p = 15$ for low dimension scenario.
* Correlated feature scenarios with correlation $\rho = 0.9$.

Table S5: RMSE comparison for high dimensional datasets shown in Figure 3(B).

Dataset	VarGuid	Lasso	FMRS	Random Forest	BART	Gradient Boosting	XGBoost	LightGBM	CatBoost
Alon	0.522	0.520	0.893	0.879	0.683	0.652	0.646	0.647	0.647
Christensen	0.094	0.110	0.633	0.481	0.370	0.356	0.350	0.345	0.345
Gravier	0.300	0.305	0.675	0.507	0.407	0.392	0.389	0.398	0.398
Pomeroy	0.564	0.892	0.882	0.611	0.540	0.627	0.549	0.544	0.544
Shipp	1.075	1.168	0.936	0.657	0.600	0.569	0.593	0.580	0.580
Singh	1.088	1.095	0.994	0.683	0.636	0.598	0.617	0.612	0.612
Tian	0.567	1.039	0.980	0.720	0.678	0.641	0.658	0.656	0.656
West	0.522	0.821	0.960	0.568	0.597	0.586	0.537	0.615	0.615
Gordon	0.159	0.158	0.899	0.459	0.418	0.358	0.347	0.373	0.373
Subramanian	0.792	1.305	0.898	0.963	0.979	0.890	0.976	1.047	1.047
cobra2	0.341	0.428	0.755	0.684	0.680	0.684	0.707	0.783	0.783
cobra8	0.242	0.280	0.562	0.505	0.503	0.507	0.513	0.554	0.554
friedman1	0.734	2.608	2.147	1.923	1.767	1.950	1.553	1.496	1.496
friedman3	0.239	0.240	1.687	1.511	1.391	1.195	1.220	1.189	1.189
inx1	0.409	0.410	1.503	1.348	1.236	1.070	1.098	1.071	1.071
inx2	0.252	0.270	1.352	1.206	1.090	0.945	0.968	0.949	0.949
inx3	0.424	0.537	1.172	1.137	1.035	0.901	0.913	0.907	0.907
lmi2	3.228	2.499	3.141	3.819	3.779	3.770	3.833	4.176	4.176
sup	3.036	2.724	3.304	3.909	3.871	3.831	4.002	4.237	4.237
sup2	0.503	0.504	2.964	3.601	3.564	3.527	3.681	3.894	3.894
cobra2*	0.697	0.715	0.489	0.316	0.314	0.331	0.325	0.389	0.389
cobra8*	0.250	0.343	0.298	0.247	0.251	0.257	0.256	0.294	0.294
friedman1*	0.783	3.607	2.273	0.864	0.823	0.849	0.865	1.009	1.009
friedman3 *	0.286	0.295	0.778	0.713	0.688	0.706	0.717	0.855	0.855
inx1*	0.455	0.478	0.469	0.651	0.631	0.648	0.651	0.791	0.791
inx2*	0.362	0.365	0.369	0.585	0.568	0.584	0.582	0.717	0.717
inx3*	0.570	0.586	0.548	0.547	0.532	0.581	0.538	0.684	0.684
lmi2*	3.355	3.038	4.539	4.654	4.671	5.007	4.689	5.631	5.631
sup*	4.931	6.237	6.930	4.829	4.894	5.473	4.983	6.743	6.743
sup2*	2.617	2.635	3.204	4.411	4.467	4.987	4.552	6.141	6.141

† Simulated datasets – cobra2, cobra8, friedman1,friedman3, inx1, inx2, inx3,lmi2, sup and sup2 – used a sample size of $N = 100$ and $p = 200$ for high dimension scenario.
* Correlated feature scenarios with correlation $\rho = 0.9$.

References

Biau, G., Fischer, A., Guedj, B. & Malley, J. D. (2016), 'COBRA: A combined regression strategy', *Journal of Multivariate Analysis* **146**, 18–28.

Buehlmann, P. & Hothorn, T. (2007), 'Boosting algorithms: Regularization, prediction and model fitting (with discussion)', *Statistical Science* **22**(4), 477–505.

CatBoost Developers (2024), *catboost: High Performance Gradient Boosting on Decision Trees Library*. R package version 1.2.5.
URL: *https://github.com/catboost/catboost*

Dorie, V. (2025), *dbarts: Discrete Bayesian Additive Regression Trees Sampler*. R package version 0.9-32.
URL: *https://CRAN.R-project.org/package=dbarts*

Friedman, J. H. (1991), 'Multivariate adaptive regression splines', *The Annals of Statistics* **19**(1), 1–67.

Friedman, J. H. (2001), 'Greedy function approximation: a gradient boosting machine', *Annals of Statistics* **29**(5), 1189–1232.

Friedman, J. H., Hastie, T. & Tibshirani, R. (2010), 'Regularization paths for generalized linear models via coordinate descent', *Journal of statistical software* **33**, 1–22.

Hofner, B., Boccuto, L. & Goeker, M. (2015), 'Controlling false discoveries in high-dimensional situations: Boosting with stability selection', *BMC Bioinformatics* **16**(144).

Hofner, B., Mayr, A., Robinzonov, N. & Schmid, M. (2014), 'Model-based boosting in R: A hands-on tutorial using the R package mboost', *Computational Statistics* **29**, 3–35.

Hothorn, T., Buehlmann, P., Kneib, T., Schmid, M. & Hofner, B. (2010), 'Model-based boosting 2.0', *Journal of Machine Learning Research* **11**, 2109–2113.

Ridgeway, G. & Developers, G. (2024), *gbm: Generalized Boosted Regression Models*. R package version 2.2.2.
URL: *https://github.com/gbm-developers/gbm*

Wright, M. N. & Ziegler, A. (2017), 'ranger: A fast implementation of random forests for high dimensional data in C++ and R', *Journal of Statistical Software* **77**(1), 1–17.
